# A unified mechanism for proteolysis and autocatalytic activation in the 20S proteasome

**DOI:** 10.1038/ncomms10900

**Published:** 2016-03-11

**Authors:** Eva M. Huber, Wolfgang Heinemeyer, Xia Li, Cassandra S. Arendt, Mark Hochstrasser, Michael Groll

**Affiliations:** 1 Center for Integrated Protein Science at the Department Chemie, Lehrstuhl für Biochemie, Technische Universität München, Lichtenbergstrasse 4, 85747 Garching, Germany; 2 Department of Molecular Biophysics and Biochemistry, Yale University, 266 Whitney Avenue, New Haven, Connecticut 06520-8114, USA; 3 Department of Biochemistry and Molecular Biology, University of Chicago, Chicago, Illinois 60637, USA

## Abstract

Biogenesis of the 20S proteasome is tightly regulated. The N-terminal propeptides protecting the active-site threonines are autocatalytically released only on completion of assembly. However, the trigger for the self-activation and the reason for the strict conservation of threonine as the active site nucleophile remain enigmatic. Here we use mutagenesis, X-ray crystallography and biochemical assays to suggest that Lys33 initiates nucleophilic attack of the propeptide by deprotonating the Thr1 hydroxyl group and that both residues together with Asp17 are part of a catalytic triad. Substitution of Thr1 by Cys disrupts the interaction with Lys33 and inactivates the proteasome. Although a Thr1Ser mutant is active, it is less efficient compared with wild type because of the unfavourable orientation of Ser1 towards incoming substrates. This work provides insights into the basic mechanism of proteolysis and propeptide autolysis, as well as the evolutionary pressures that drove the proteasome to become a threonine protease.

## Abstract (web summary)

The proteasome, an essential molecular machine, is a threonine protease, but the evolution and the components of its proteolytic centre are unclear. Here, the authors use structural biology and biochemistry to investigate the role of proteasome active site residues on maturation and activity.

## Graphical abstract

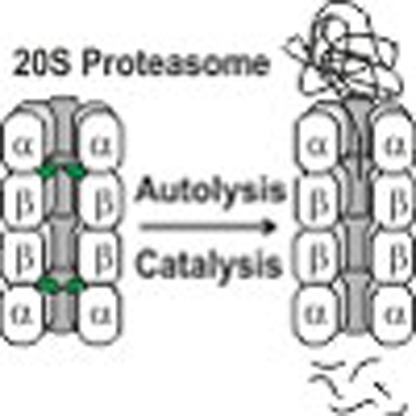

The 20S proteasome core particle (CP) is the key non-lysosomal protease of eukaryotic cells. Its seven different α and seven different β subunits assemble into four heptameric rings that are stacked on each other to form a hollow cylinder. While the inactive α subunits build the two outer rings, the β subunits form the inner rings. Only three out of the seven different β subunits, namely β1, β2 and β5, bear N-terminal proteolytic active centres, and before CP maturation these are protected by propeptides^1^,^2^,^3^. In the last stage of CP biogenesis, the prosegments are autocatalytically removed through nucleophilic attack by the active site residue Thr1 on the preceding peptide bond involving Gly(-1)^4^,^5^. Release of the propeptides creates a functionally active CP that cleaves proteins into short peptides.

Although the chemical nature of the substrate-binding channel and hence substrate preferences are unique to each of the distinct active β subunits^6^,^7^, all active sites employ an identical reaction mechanism to hydrolyse peptide bonds^2^. Nucleophilic attack of Thr1O^γ^ on the carbonyl carbon atom of the scissile peptide bond creates a first cleavage product and a covalent acyl-enzyme intermediate. Hydrolysis of this complex by the addition of a nucleophilic water molecule regenerates the enzyme and releases the second peptide fragment^8^,^9^. The proteasome belongs to the family of N-terminal nucleophilic (Ntn) hydrolases^10^, and the free N-terminal amine group of Thr1 was proposed to deprotonate the Thr1 hydroxyl group to generate a nucleophilic Thr1O^γ^ for peptide-bond cleavage^2^,^9^,^11^. This mechanism, however, cannot explain autocatalytic precursor processing because in the immature active sites, Thr1N is part of the peptide bond with Gly(-1), the bond that needs to be hydrolysed. An alternative candidate for deprotonating the Thr1 hydroxyl group is the side chain of Lys33 as it is within hydrogen-bonding distance to Thr1OH (2.7 Å). In principle it could function as the general base during both autocatalytic removal of the propeptide and protein substrate cleavage. Here we provide experimental evidences for this distinct view of the proteasome active-site mechanism. Data from biochemical and structural analyses of proteasome variants with mutations in the β5 propeptide and the active site strongly support the model and deliver novel insights into the structural constraints required for the autocatalytic activation of the proteasome. Furthermore, we determine the advantages of Thr over Cys or Ser as the active-site nucleophile using X-ray crystallography together with activity and inhibition assays.

## Results

### Inactivation of proteasome subunits by T1A mutations

Proteasome-mediated degradation of cell-cycle regulators and potentially toxic misfolded proteins is required for the viability of eukaryotic cells^8^. Inactivation of the active site Thr1 by mutation to Ala has been used to study substrate specificity and the hierarchy of the proteasome active sites^1^,^4^,^12^,^13^,^14^,^15^. Yeast strains carrying the single mutations β1-T1A or β2-T1A, or both, are viable, even though one or two of the three distinct catalytic β subunits are disabled and carry remnants of their N-terminal propeptides^4^ (Table 1). These results indicate that the β1 and β2 proteolytic activities are not essential for cell survival. By contrast, the T1A mutation in subunit β5 has been reported to be lethal or nearly so^1^,^13^. Viability is restored if the β5-T1A subunit has its propeptide (pp) deleted but expressed separately *in trans* (β5-T1A pp *trans*), although substantial phenotypic impairment remains^1^,^15^,^16^ (Table 1). Our present crystallographic analysis of the β5-T1A pp *trans* mutant demonstrates that the mutation *per se* does not structurally alter the catalytic active site and that the *trans*-expressed β5 propeptide is not bound in the β5 substrate-binding channel (Supplementary Fig. 1a).

The extremely weak growth of the β5-T1A mutant pp *cis* described by Chen and Hochstrasser^1^ compared with the inviability reported by Heinemeyer *et al*.^13^ prompted us to analyse this discrepancy. Sequencing of the plasmids, testing them in both published yeast strain backgrounds and site-directed mutagenesis revealed that the β5-T1A mutant pp *cis* is viable, but suffers from a marked growth defect that requires extended incubation of 4–5 days for initial colony formation (Table 1 and Supplementary Methods). We also identified an additional point mutation K81R in subunit β5 that was present in the allele used in ref. 1. This single amino-acid exchange is located at the interface of the subunits α4, β4 and β5 (Supplementary Fig. 1b) and might weakly promote CP assembly by enhancing inter-subunit contacts. The slightly better growth of the β5-T1A-K81R mutant allowed us to solve the crystal structure of a yeast proteasome (yCP) with the β5-T1A mutation, which is discussed in the following section (for details see Supplementary Note 1).

### Propeptide conformation and triggering of autolysis

**Figure 1:**
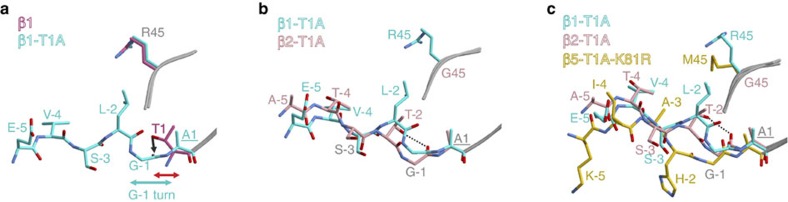
Conformation of proteasomal propeptides. (**a**) Structural superposition of the β1-T1A propeptide and the matured WT β1 active-site Thr1. Only the residues (-5) to (-1) of the β1-T1A propeptide are displayed. The major determinant of the S1 specificity pocket, residue 45, is depicted. Note the tight conformation of Gly(-1) and Ala1 before propeptide removal (G(-1) turn; cyan double arrow) compared with the relaxed, processed WT active-site Thr1 (red double arrow). The black arrow indicates the attack of Thr1O^γ^ onto the carbonyl carbon atom of Gly(-1). (**b**) Structural superposition of the β1-T1A propeptide and the β2-T1A propeptide highlights subtle differences in their conformations, but illustrates that Ala1 and Gly(-1) match well. Thr(-2)OH is hydrogen-bonded to Gly(-1)O (∼2.8 Å; black dashed line). The major determinant of the S1 specificity pocket, residue 45, is depicted. (**c**) Structural superposition of the β1-T1A, the β2-T1A and the β5-T1A-K81R propeptide remnants depict their differences in conformation. While residue (-2) of the β1 and β2 prosegments fit the S1 pocket, His(-2) of the β5 propeptide occupies the S2 pocket. Nonetheless, in all mutants the carbonyl carbon atom of Gly(-1) is ideally placed for the nucleophilic attack by Thr1O^γ^. The hydrogen bond between Thr(-2)OH and Gly(-1)O (∼2.8 Å) is indicated by a black dashed line.

In the final steps of proteasome biogenesis, the propeptides are autocatalytically cleaved from the mature β-subunit domains^1^. For subunit β1, this process was previously inferred to require that the propeptide residue at position (-2) of the subunit precursor occupies the S1 specificity pocket of the substrate-binding channel formed by amino acid 45 (for details see Supplementary Note 2)^5^. Furthermore, it was observed that the prosegment forms an antiparallel β-sheet in the active site, and that Gly(-1) adopts a γ-turn conformation, which by definition is characterized by a hydrogen bond between Leu(-2)O and Thr1NH (ref. 5). Here we again analysed the β1-T1A mutant crystallographically but in addition determined the structures of the β2-T1A single and β1-T1A-β2-T1A double mutants (Protein Data Bank (PDB) entry codes are provided in Supplementary Table 1). In subunit β1, we found that Gly(-1) indeed forms a sharp turn, which relaxes on prosegment cleavage (Fig. 1a and Supplementary Fig. 2a). However, the γ-turn conformation and the associated hydrogen bond initially proposed is for geometric and chemical reasons inappropriate and would not perfectly position the carbonyl carbon atom of Gly(-1) for nucleophilic attack by Thr1. Regarding the β2 propeptide, Thr(-2) occupies the S1 pocket but is less deeply anchored compared with Leu(-2) in β1, which might be due to the rather large β2-S1 pocket created by Gly45. Thr(-2) positions Gly(-1)O via hydrogen bonding (∼2.8 Å) in a perfect trajectory for the nucleophilic attack by Thr1O^γ^ (Fig. 1b and Supplementary Fig. 2b). Next, we examined the position of the β5 propeptide in the β5-T1A-K81R mutant. Surprisingly, Gly(-1) is completely extended and forces the histidine side chain at position (-2) to occupy the S2 instead of the S1 pocket, thereby disrupting the antiparallel β-sheet. Nonetheless, the carbonyl carbon of Gly(-1) would be ideally placed for nucleophilic attack by Thr1O^γ^ (Fig. 1c and Supplementary Fig. 2c,d). As the K81R mutation is located far from the active site (Thr1C^α^–Arg81C^α^: 24 Å), any influence on propeptide conformation can be excluded. Instead, the plasticity of the β5 S1 pocket caused by the rotational flexibility of Met45 might prevent stable accommodation of His(-2) in the S1 site and thus also promote its immediate release after autolysis.

Processing of β-subunit precursors requires deprotonation of Thr1OH; however, the general base initiating autolysis is unknown. Remarkably, eukaryotic proteasomal β5 subunits bear a His residue in position (-2) of the propeptide (Supplementary Fig. 3a). As histidine commonly functions as a proton shuttle in the catalytic triads of serine proteases^17^, we investigated the role of His(-2) in processing of the β5 propeptide by exchanging it for Asn, Lys, Phe and Ala. All yeast mutants were viable at 30 °C, but suffered from growth defects at 37 °C with the H(-2)N and H(-2)F mutants being most affected (Supplementary Fig. 3b and Table 1). In agreement, the chymotrypsin-like (ChT-L) activity of H(-2)N and H(-2)F mutant yCPs was impaired *in situ* and *in vitro* (Supplementary Fig. 3c). Structural analyses revealed that the propeptides of all mutant yCPs shared residual 2*F*_O_–*F*_C_ electron densities. Gly(-1) and Phe/Lys(-2) were visualized at low occupancy, while Ala/Asn(-2) could not be assigned. This observation indicates a mixture of processed and unprocessed β5 subunits and partially impaired autolysis^18^, thereby excluding any essential role of residue (-2) as the general base.

**Figure 2:**
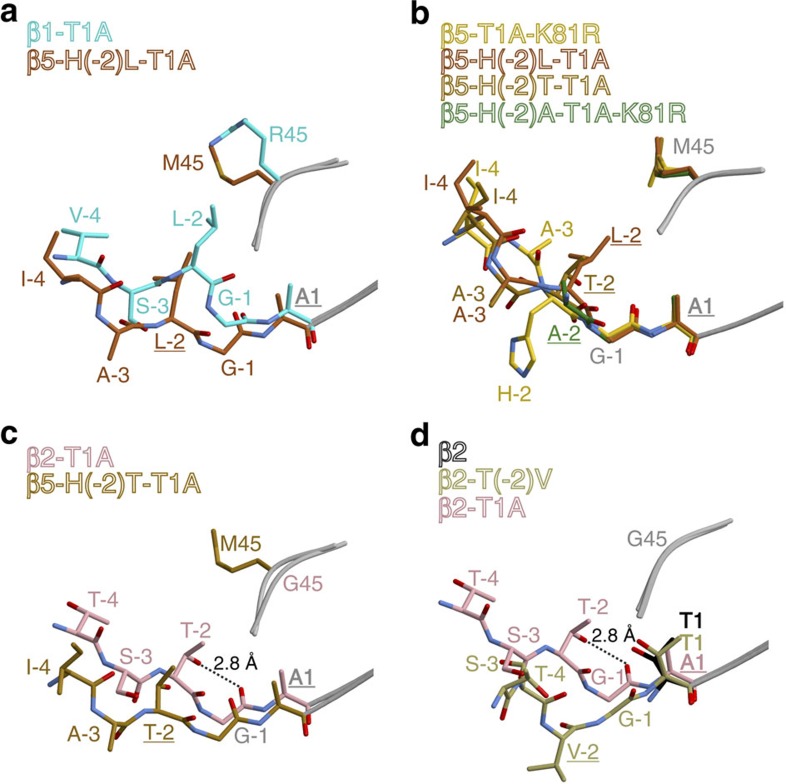
Mutations of residue (-2) and their influence on propeptide conformation and autolysis. (**a**) Structural superposition of the β1-T1A propeptide and the β5-H(-2)L-T1A mutant propeptide. The (-2) residues of both prosegments point into the S1 pocket. (**b**) Structural superposition of the β5 propeptides in the β5-H(-2)L-T1A, β5-H(-2)T-T1A, β5-(H-2)A-T1A-K81R and β5-T1A-K81R mutant proteasomes. While the residues (-2) to (-4) vary in their conformation, Gly(-1) and Ala1 are located in all structures at the same positions. (**c**) Structural superposition of the β2-T1A propeptide and the β5-H(-2)T-T1A mutant propeptide. The (-2) residues of both prosegments point into the S1 pocket, but only Thr(-2)OH of β2 forms a hydrogen bridge to Gly(-1)O (black dashed line). (**d**) Structural superposition of the matured β2 active site, the WT β2-T1A propeptide and the β2-T(-2)V mutant propeptide. Notably, Val(-2) of the latter does not occupy the S1 pocket, thereby changing the orientation of Gly(-1) and preventing nucleophilic attack of Thr1O^γ^ on the carbonyl carbon atom of Gly(-1). For all panels stereo views are provided in Supplementary Fig. 4g–j.

Next, we examined the effect of residue (-2) on the orientation of the propeptide by creating mutants that combine the T1A (K81R) mutation(s) with H(-2)L, H(-2)T or H(-2)A substitutions. Leu(-2) is encoded in the yeast β1 subunit precursor (Supplementary Fig. 3a); Thr(-2) is generally part of β2-propeptides (Supplementary Fig. 3a); and Ala(-2) was expected to fit the β5-S1 pocket without inducing conformational changes of Met45, allowing it to accommodate ‘β1-like' propeptide positioning. As expected from β5-T1A mutants, the yeasts show severe growth phenotypes, with minor variations (Supplementary Fig. 4a and Table 1). We determined crystal structures of the β5-H(-2)L-T1A, β5-H(-2)T-T1A and the β5-H(-2)A-T1A-K81R mutants (Supplementary Table 1). For the β5-H(-2)A-T1A-K81R variant, only the residues Gly(-1) and Ala(-2) could be visualized, indicating that Ala(-2) leads to insufficient stabilization of the propeptide in the substrate-binding channel (Supplementary Fig. 4d). By contrast, the prosegments of the β5-H(-2)L-T1A and the β5-H(-2)T-T1A mutants were significantly better resolved in the 2*F*_O_–*F*_C_ electron-density maps yet not at full occupancy (Supplementary Fig. 4b,c and Supplementary Table 1), suggesting that the natural propeptide bearing His(-2) is most favourable. Nevertheless, both Leu(-2) and Thr(-2) were found to occupy the S1 specificity pocket formed by Met45 (Fig. 2a,b and Supplementary Fig. 4f–h). This result proves that the naturally occurring His(-2) of the β5 propeptide does not stably fit into the S1 site. Since Gly(-1) adopts the same position in both wild-type (WT) and mutant β5 propeptides, and since in all cases its carbonyl carbon is perfectly placed for nucleophilic attack by Thr1O^γ^ (Fig. 2b), we propose that neither binding of residue (-2) to the S1 pocket nor formation of the antiparallel β-sheet is essential for autolysis of the propeptide.

Next, we determined the crystal structure of a chimeric yCP having the yeast β1-propeptide replaced by its β5 counterpart^18^. Although we observed fragments of 2*F*_O_–*F*_C_ electron density in the β1 active site, the data were not interpretable. Bearing in mind that in contrast to Thr(-2) in β2, Leu(-2) in subunit β1 is not conserved among species (Supplementary Fig. 3a), we created a β2-T(-2)V proteasome mutant. As proven by the β2-T1A crystal structures, Thr(-2) hydrogen bonds to Gly(-1)O. Although this interaction was not observed for the β5-H(-2)T-T1A mutant (Fig. 2c and Supplementary Fig. 4c,i), exchange of Thr(-2) by Val in β2, a conservative mutation regarding size but drastic with respect to polarity, was found to inhibit maturation of this subunit (Fig. 2d and Supplementary Fig. 4e,j). Notably, the 2*F*_O_–*F*_C_ electron-density map displays a different orientation for the β2 propeptide than has been observed for the β2-T1A proteasome. In particular, Val(-2) is displaced from the S1 site and Gly(-1) is severely shifted (movement of the carbonyl oxygen atom of 3.8 Å), thereby preventing nucleophilic attack of Thr1 (Fig. 2d and Supplementary Fig. 4j,k). These results further confirm that correct positioning of the active-site residues and Gly(-1) is decisive for the maturation of the proteasome.

### The active site of the proteasome

**Figure 3:**
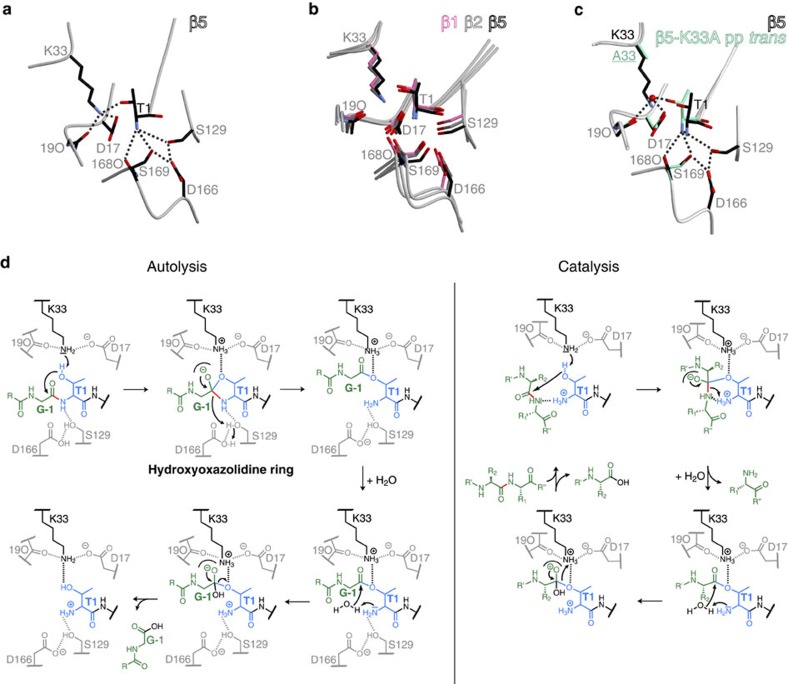
Architecture and proposed reaction mechanism of the proteasomal active site. (**a**) Hydrogen-bonding network at the mature WT β5 proteasomal active site (dotted lines). Thr1OH is hydrogen-bonded to Lys33NH_2_ (2.7 Å), which in turn interacts with Asp17O^δ^. The Thr1 N terminus is engaged in hydrogen bonds with Ser129O^γ^, the carbonyl oxygen of residue 168, Ser169O^γ^ and Asp166O^δ^. (**b**) The orientations of the active-site residues involved in hydrogen bonding are strictly conserved in each proteolytic centre, as shown by superposition of the β subunits. (**c**) Structural superposition of the WT β5 and the β5-K33A pp *trans* mutant active site. In the latter, a water molecule (red sphere) is found at the position where in the WT structure the side chain amine group of Lys33 is located. Similarly to Lys33, the water molecule hydrogen bonds to Arg19O, Asp17O^δ^ and Thr1OH. Note, the strong interaction with the water molecule causes a minor shift of Thr1, while all other active-site residues remain in place. (**d**) Proposed chemical reaction mechanism for autocatalytic precursor processing and proteolysis in the proteasome. The active-site Thr1 is depicted in blue, the propeptide segment and the peptide substrate are coloured in green, whereas the scissile peptide bond is highlighted in red. Autolysis (left set of structures) is initiated by deprotonation of Thr1OH via Lys33NH_2_ and the formation of a tetrahedral transition state. The strictly conserved oxyanion hole Gly47NH stabilizing the negatively charged intermediate is illustrated as a semicircle. Collapse of the transition state frees the Thr1 N terminus (by completing an N-to-O acyl shift of the propeptide), which is subsequently protonated by Asp166OH via Ser129OH. Next, Thr1NH_2_ polarizes a water molecule for the nucleophilic attack of the acyl-enzyme intermediate. On hydrolysis of the latter, the active-site Thr1 is ready for catalysis (right set of structures). Substrate processing starts with nucleophilic attack of the carbonyl carbon atom of the scissile peptide bond. The charged Thr1 N terminus may engage in the orientation of the amide moiety and donate a proton to the emerging N terminus of the C-terminal cleavage product. The resulting deprotonated Thr1NH_2_ finally activates a water molecule for hydrolysis of the acyl-enzyme.

**Figure 4:**
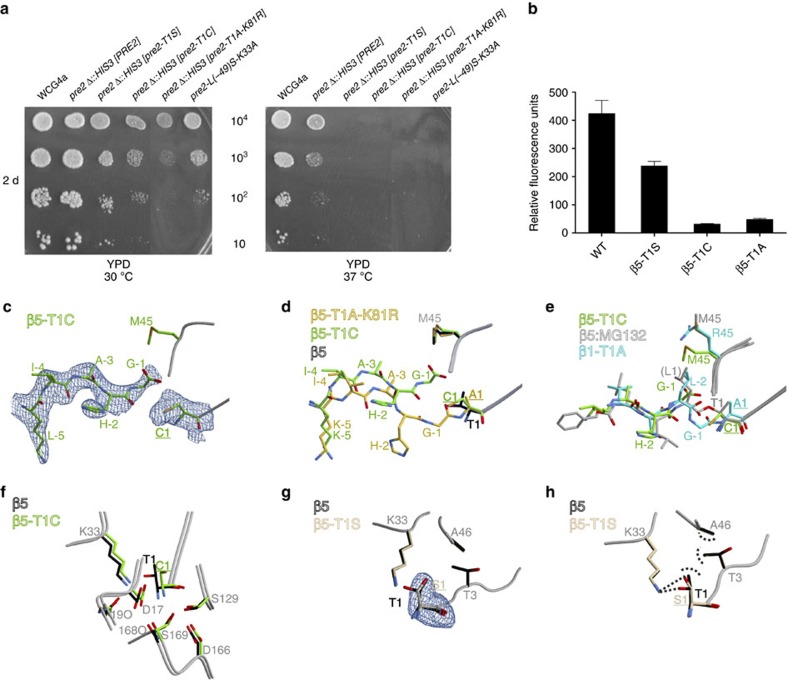
The proteasome favours threonine as the active-site nucleophile. (**a**) Growth tests by serial dilution of WT and *pre2* (β5) mutant yeast cultures reveal growth defects of the active-site mutants under the indicated conditions after 2 days (2 d) of incubation. (**b**) Purified WT and mutant proteasomes were tested for their chymotrypsin-like activity (β5) using the substrate Suc-LLVY-AMC. Relative fluorescence units were measured in triplicate after 1 h of incubation at room temperature and are given as mean values. S.d.'s are indicated by error bars. (**c**) Illustration of the 2*F*_O_–*F*_C_ electron-density map (blue mesh contoured at 1*σ*) for the β5-T1C propeptide fragment. The prosegment is cleaved but still bound in the substrate-binding channel. Notably, His(-2) does not occupy the S1 pocket formed by Met45, similar to what was observed for the β5-T1A-K81R mutant. (**d**) Structural superposition of the β5-T1A-K81R and the β5-T1C mutant subunits onto the WT β5 subunit. (**e**) Structural superposition of the β5-T1C propeptide onto the β1-T1A active site (blue) and the WT β5 active site in complex with the proteasome inhibitor MG132 (ref. 30). The inhibitor as well as the propeptides adopt similar conformations in the substrate-binding channel. (**f**) Structural superposition of the WT β5 and β5-T1C mutant active sites illustrates the different orientations of the hydroxyl group of Thr1 and the thiol side chain of Cys1. The SH group is rotated by 74° compared with the OH group. (**g**) Structural superposition of the WT β5 and β5-T1S mutant active sites reveals different orientations of the hydroxyl groups of Thr1 and Ser1, respectively. The 2*F*_O_–*F*_C_ electron-density map for Ser1 (blue mesh contoured at 1*σ*) is illustrated. (**h**) The methyl group of Thr1 is anchored by hydrophobic interactions with Ala46C^β^ and Thr3C^γ^. Ser1 lacks this stabilization and is therefore rotated by 60°.

Proton shuttling from the proteasomal active site Thr1OH to Thr1NH_2_ via a nucleophilic water molecule was suggested to initiate peptide-bond hydrolysis^2^,^9^,^10^. However, in the immature particle Thr1NH_2_ is blocked by the propeptide and cannot activate Thr1O^γ^. Instead, Lys33NH_2_, which is in hydrogen-bonding distance to Thr1O^γ^ (2.7 Å) in all catalytically active β subunits (Fig. 3a,b)^2^,^9^, was proposed to serve as the proton acceptor^19^. Owing to its likely protonation at neutral pH, however, it was assumed not to act as the general base^2^,^5^,^9^. A proposed catalytic tetrad model involving Thr1OH, Thr1NH_2_, Lys33NH_2_ and Asp17O^δ^, as well as a nucleophilic water molecule as the proton shuttle appeared to accommodate all possible views of the proteasomal active site^8^,^9^,^20^. Twenty years later, with a plethora of yCP X-ray structures in hand, we decided to re-analyse the active site of the proteasome and to resolve the uncertainty regarding the nature of the general base. Mutation of β5-Lys33 to Ala causes a strongly deleterious phenotype, and previous structural and biochemical analyses confirmed that this is caused by failure of propeptide cleavage, and consequently, lack of ChT-L activity^1^,^4^,^13^ (Fig. 4a, Supplementary Fig. 3b and Table 1; for details see Supplementary Note 1). The phenotype of the β5-K33A mutant was however less pronounced than for the β5-T1A-K81R yeast (Fig. 4a). This discrepancy in growth was traced to an additional point mutation L(-49)S in the β5-propeptide of the β5-K33A mutant (see also Supplementary Note 1). Structural comparison of the β5-L(-49)S-K33A and β5-T1A-K81R active sites revealed that mutation of Lys33 to Ala creates a cavity that is filled with Thr1 and the remnant propeptide. This structural alteration destroys active-site integrity and abolishes catalytic activity of the β5 active site^4^ (Supplementary Fig. 5a). Additional proof for the key function of Lys33 was obtained from the β5-K33A mutant, with the propeptide expressed separately from the main subunit (pp *trans*)^15^. The Thr1 N terminus of this mutant is not blocked by the propeptide, yet its catalytic activity is reduced by ∼83% (Supplementary Fig. 6b). Consistent with this, the crystal structure of the β5-K33A pp *trans* mutant in complex with carfilzomib only showed partial occupancy of the ligand at the β5 active sites (Supplementary Fig. 5b and Supplementary Table 1). Since no acetylation of the Thr1 N terminus was observed for the β5-K33A pp *trans* apo crystal structure^4^,^16^, the reduced reactivity towards substrates and inhibitors indicates that Lys33NH_2_, rather than Thr1NH_2_, deprotonates and activates Thr1OH. Furthermore, the crystal structure of the β5-K33A pp *trans* mutant without inhibitor revealed that Thr1O^γ^ strongly coordinates a well-defined water molecule (∼2 Å; Fig. 3c and Supplementary Fig. 5c,d). This water hydrogen bonds also to Arg19O (∼3.0 Å) and Asp17O^δ^ (∼3.0 Å), and thereby presumably enables residual activity of the mutant. Remarkably, the solvent molecule occupies the position normally taken by Lys33NH_2_ in the WT proteasome structure (Fig. 3c), further corroborating the essential role of Lys33 as the general base for autolysis and proteolysis. Conservative substitution of Lys33 by Arg delays autolysis of the β5 precursor and impairs yeast growth (for details see Supplementary Note 1). While Thr1 occupies the same position as in WT yCPs, Arg33 is unable to hydrogen bond to Asp17, thereby inactivating the β5 active site^2^,^4^ (Supplementary Fig. 5e).

The conservative mutation of Asp17 to Asn in subunit β5 of the yCP also provokes a severe growth defect (Supplementary Note 1, Supplementary Fig. 6a and Table 1). Notably, only with the additional point mutation L(-49)S present in the β5 propeptide could we purify a small amount of the β5-D17N mutant yCP. As determined by crystallographic analysis, this mutant β5 subunit was partially processed (Table 1) but displayed impaired reactivity towards the proteasome inhibitor carfilzomib compared with the subunits β1 and β2, and with WT β5 (Supplementary Fig. 7a). In contrast to the *cis*-construct, expression of the β5 propeptide *in trans* allowed straightforward isolation and crystallization of the D17N mutant proteasome. The ChT-L activity of the β5-D17N pp *in trans* CP towards the canonical β5 model substrates *N*-succinyl-Leu-Leu-Val-Tyr-7-amino-4-methylcoumarin (Suc-LLVY-AMC) and carboxybenzyl-Gly-Gly-Leu-para-nitroanilide (Z-GGL-pNA) was severely reduced (Supplementary Fig. 6b), confirming that Asp17 is of fundamental importance for the catalytic activity of the mature proteasome. Even though the β5-D17N pp *trans* yCP crystal structure appeared identical to the WT yCP (Supplementary Fig. 7b), the co-crystal structure with the α′, β′ epoxyketone inhibitor carfilzomib visualized only partial occupancy of the ligand in the β5 active site (Supplementary Fig. 7a). This observation is consistent with a strongly reduced reactivity of β5-Thr1 and the crystal structure of the β5-D17N pp *cis* mutant in complex with carfilzomib. Autolysis and residual catalytic activity of the β5-D17N mutants may originate from the carbonyl group of Asn17, which albeit to a lower degree still can polarize Lys33 for the activation of Thr1. In agreement, an E17A mutant in the proteasomal β-subunit of the archaeon *Thermoplasma acidophilum* prevents autolysis and catalysis^21^. Strikingly, although the X-ray data on the β5-D17N mutant with the propeptide expressed in *cis* and in *trans* looked similar, there was a pronounced difference in their growth phenotypes observed (Supplementary Fig. 6a and Supplementary Fig. 7b).

On the basis of these results, we propose that CPs from all domains of life use a catalytic triad consisting of Thr1, Lys33 and Asp/Glu17 for both autocatalytic precursor processing and proteolysis (Fig. 3d). This model is also consistent with the fact that no defined water molecule is observed in the mature WT proteasomal active site that could shuttle the proton from Thr1O^γ^ to Thr1NH_2_.

To explore this active-site model further, we exchanged the conserved Asp166 residue for Asn in the yeast β5 subunit. Asp166O^δ^ is hydrogen-bonded to Thr1NH_2_ via Ser129OH and Ser169OH, and therefore was proposed to be involved in catalysis^2^. The β5-D166N pp *cis* yeast mutant is significantly impaired in growth and its ChT-L activity is drastically reduced (Supplementary Fig. 6a,b and Table 1). X-ray data on the β5-D166N mutant indicate that the β5 propeptide is hydrolysed, but due to reorientation of Ser129OH, the interaction with Asn166O^δ^ is disrupted (Supplementary Fig. 8a). Instead, a water molecule is bound to Ser129OH and Thr1NH_2_ (Supplementary Fig. 8b), which may enable precursor processing. The hydrogen bonds involving Ser169OH are intact and may account for residual substrate turnover. Soaking the β5-D166N crystals with carfilzomib and MG132 resulted in covalent modification of Thr1 at high occupancy (Supplementary Fig. 8c). In the carfilzomib complex structure, Thr1O^γ^ and Thr1N incorporate into a morpholine ring structure and Ser129 adopts its WT-like orientation. In the MG132-bound state, Thr1N is unmodified, and we again observe that Ser129 is hydrogen-bonded to a water molecule instead of Asn166. Whereas Asn can to some degree replace Asp166 due to its carbonyl group in the side chain, Ala at this position was found to prevent both autolysis and catalysis^21^. These results suggest that Asp166 and Ser129 function as a proton shuttle and affect the protonation state of Thr1N during autolysis and catalysis.

### Substitution of the active-site Thr1 by Cys

Mutation of Thr1 to Cys inactivates the 20S proteasome from the archaeon *T. acidophilum*^21^. In yeast, this mutation causes a strong growth defect (Fig. 4a and Table 1), although the propeptide is hydrolysed, as shown here by its X-ray structure. In one of the two β5 subunits, however, we found the cleaved propeptide still bound in the substrate-binding channel (Fig. 4c). His(-2) occupies the S2 pocket like observed for the β5-T1A-K81R mutant, but in contrast to the latter, the propeptide in the T1C mutant adopts an antiparallel β-sheet conformation as known from inhibitors like MG132 (Fig. 4c–e and Supplementary Fig. 9b). On the basis of the phenotype of the T1C mutant and the propeptide remnant identified in its active site, we suppose that autolysis is retarded and may not have been completed before crystallization. Owing to the unequal positions of the two β5 subunits within the CP in the crystal lattice, maturation and propeptide displacement may occur at different timescales in the two subunits.

Despite propeptide hydrolysis, the β5-T1C active site is catalytically inactive (Fig. 4b and Supplementary Fig. 9a). In agreement, soaking crystals with the CP inhibitors bortezomib or carfilzomib modifies only the β1 and β2 active sites, while leaving the β5-T1C proteolytic centres unmodified even though they are only partially occupied by the cleaved propeptide remnant. Moreover, the structural data reveal that the thiol group of Cys1 is rotated by 74° with respect to the hydroxyl side chain of Thr1 (Fig. 4f and Supplementary Fig. 9b). This presumably results from the larger radius of the sulfur atom compared with oxygen. Consequently, the hydrogen bond bridging the active-site nucleophile and Lys33 in WT CPs is broken with Cys1. Notably, the 2*F*_O_–*F*_C_ electron-density map of the T1C mutant also indicates that Lys33NH_2_ is disordered. Together, these observations suggest that efficient peptide-bond hydrolysis requires that Lys33NH_2_ hydrogen bonds to the active site nucleophile.

### The benefit of Thr over Ser as the active-site nucleophile

**Figure 5:**
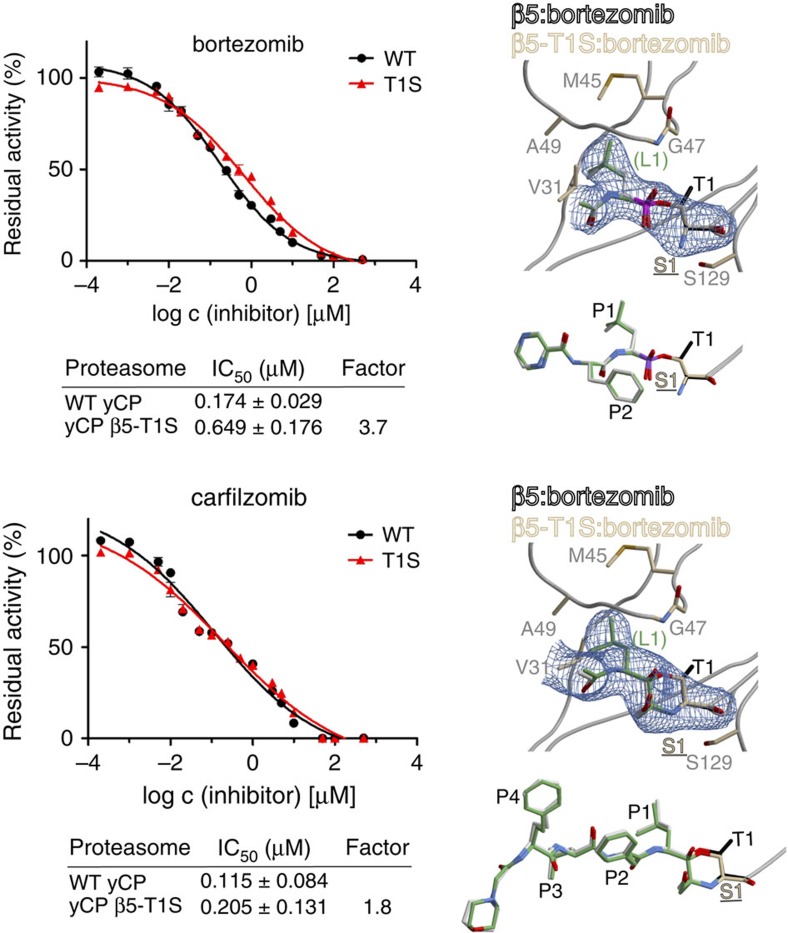
Inhibition of WT and mutant β5-T1S proteasomes by bortezomib and carfilzomib. Inhibition assays (left panel). Purified yeast proteasomes were tested for the susceptibility of their ChT-L (β5) activity to inhibition by bortezomib and carfilzomib using the substrate Suc-LLVY-AMC. IC_50_ values were determined in triplicate; s.d.'s are indicated by error bars. Note that IC_50_ values depend on time and enzyme concentration. Proteasomes (final concentration: 66 nM) were incubated with inhibitor for 45 min before substrate addition (final concentration: 200 μM). Structures of the β5-T1S mutant in complex with both ligands (green) prove the reactivity of Ser1 (right panel). The 2*F*_O_–*F*_C_ electron-density maps (blue mesh) for Ser1 (brown) and the covalently bound ligands (green; only the P1 site (Leu1) is shown) are contoured at 1*σ*. The WT proteasome:inhibitor complex structures (inhibitor in grey; Thr1 in black) are superimposed and demonstrate that mutation of Thr1 to Ser does not affect the binding mode of bortezomib or carfilzomib.

All proteasomes strictly employ threonine as the active-site residue instead of serine. To investigate the reason for this singularity, we analysed a β5-T1S mutant, which is viable but suffers from growth defects (Fig. 4a and Table 1). Activity assays with the β5-specific substrate Suc-LLVY-AMC demonstrated that the ChT-L activity of the T1S mutant is reduced by 40–45% compared with WT proteasomes depending on the incubation temperature (Fig. 4b and Supplementary Fig. 9c). By contrast, turnover of the substrate Z-GGL-pNA, used to monitor ChT-L activity *in situ* but in a less quantitative fashion, is not detectably impaired (Supplementary Fig. 9a). Crystal structure analysis of the β5-T1S mutant confirmed precursor processing (Fig. 4g), and ligand-complex structures with bortezomib and carfilzomib unambiguously corroborated the reactivity of Ser1 (Fig. 5).

However, the apo crystal structure revealed that Ser1O^γ^ is turned away from the substrate-binding channel (Fig. 4g). Compared with Thr1O^γ^ in WT CP structures, Ser1O^γ^ is rotated by 60°. This renders it unavailable for direct nucleophilic attack onto incoming substrates and first requires its reorientation, which is expected to delay substrate turnover. Because both conformations of Ser1O^γ^ are hydrogen-bonded to Lys33NH_2_ (Fig. 4h), the relay system is capable of hydrolysing peptide substrates, albeit at lower rates compared with Thr1. The active-site residue Thr1 is fixed in its position, as its methyl group is engaged in hydrophobic interactions with Thr3 and Ala46 (Fig. 4h). Consequently, the hydroxyl group of Thr1 requires no reorientation before substrate cleavage and is thus more catalytically efficient than Ser1. In agreement, at an elevated growing temperature of 37 °C the T1S mutant is unable to grow (Fig. 4a). *In vitro*, the mutant proteasome is less susceptible to proteasome inhibition by bortezomib (3.7-fold) and carfilzomib (1.8-fold; Fig. 5). Nevertheless, inhibitor complex structures indicate identical binding modes compared with the WT yCP structures, with the same inhibitors^22^,^23^. Notably, the affinity of the tetrapeptide carfilzomib is less impaired, as it is better stabilized in the substrate-binding channel than the dipeptide bortezomib, which lacks a defined P3 site and has only a few interactions with the surrounding protein. Hence, the mean residence time of carfilzomib at the active site is prolonged and the probability to covalently react with Ser1 is increased. Considered together, these results provide a plausible explanation for the invariance of threonine as the active-site nucleophile in proteasomes in all three domains of life, as well as in proteasome-like proteases such as HslV (ref. 24).

## Discussion

The 20S proteasome CP is the major non-lysosomal protease in eukaryotic cells, and its assembly is highly organized. The β-subunit propeptides, particularly that of β5, are key factors that help drive proper assembly of the CP complex^1^. In addition, they prevent irreversible inactivation of the Thr1 N terminus by *N*-acetylation^4^,^15^,^16^. By contrast, the prosegments of β subunits are dispensable for archaeal proteasome assembly, at least when heterologously expressed in *Escherichia coli*^25^. In eukaryotes, deletion of or failure to cleave the β1 and β2 propeptides is well tolerated^5^,^13^,^14^,^15^,^16^. However, removal of the β5 prosegment or any interference with its cleavage causes severe phenotypic defects^1^,^13^. These observations highlight the unique function and importance of the β5 propeptide as well as the β5 active site for maturation and function of the eukaryotic CP.

Here we have described the atomic structures of various β5-T1A mutants, which allowed for the first time visualization of the residual β5 propeptide. Depending on the (-2) residue we observed various propeptide conformations, but Gly(-1) is in all structures perfectly located for the nucleophilic attack by Thr1O^γ^, although it does not adopt the tight turn observed for the prosegment of subunit β1. From these data we conclude that only the positioning of Gly(-1) and Thr1 as well as the integrity of the proteasomal active site are required for autolysis. In this regard, inappropriate *N*-acetylation of the Thr1 N terminus cannot be removed by Thr1O^γ^ due to the rotational freedom and flexibility of the acetyl group. The propeptide needs some anchoring in the substrate-binding channel to properly position Gly(-1), but this seems to be independent of the orientation of residue (-2).

Autolytic activation of the CP constitutes one of the final steps of proteasome biogenesis^26^, but the trigger for propeptide cleavage had remained enigmatic. On the basis of the numerous CP:ligand complexes solved during the past 18 years and in the current study, we provide a revised interpretation of proteasome active-site architecture. We propose a catalytic triad for the active site of the CP consisting of residues Thr1, Lys33 and Asp/Glu17, which are conserved among all proteolytically active eukaryotic, bacterial and archaeal proteasome subunits. Lys33NH_2_ is expected to act as the proton acceptor during autocatalytic removal of the propeptides^19^, as well as during substrate proteolysis, while Asp17O^δ^ orients Lys33NH_2_ and makes it more prone to protonation by raising its p*K*_a_ (hydrogen bond distance: Lys33NH_3_^+^–Asp17O^δ^: 2.9 Å). Analogously to the proteasome, a Thr–Lys–Asp triad is also found in L-asparaginase^27^. Thus, specific protein surroundings can significantly alter the chemical properties of amino acids such as Lys to function as an acid–base catalyst^28^.

In this new view of the proteasomal active site, the positively charged Thr1NH_3_^+^-terminus hydrogen bonds to the amide nitrogen of incoming peptide substrates and stabilizes as well as activates them for the endoproteolytic cleavage by Thr1O^γ^ (Fig. 3d). Consistent with this model, the positively charged Thr1 N terminus is engaged in hydrogen bonds with inhibitory compounds like fellutamide B (ref. 29), α-ketoamides^30^, homobelactosin C (ref. 31) and salinosporamide A (ref. 32). Furthermore, opening of the β-lactone compound omuralide^2^ by Thr1 creates a C3-hydroxyl group, whose proton originates from Thr1NH_3_^+^. The resulting uncharged Thr1NH_2_ is hydrogen-bridged to the C3-OH group. In agreement, acetylation of the Thr1 N terminus irreversibly blocks hydrolytic activity^15^,^16^, and binding of substrates is prevented for steric reasons. By acting as a proton donor during catalysis, the Thr1 N terminus may also favour cleavage of substrate peptide bonds (Fig. 3d). In all proteases, collapse of the tetrahedral transition state results in selective breakage of the substrate amide bond, while the covalent interaction between the substrate and the enzyme persists. Cleavage of the scissile peptide bond requires protonation of the emerging free amine, and in the proteasome, the Thr1 amine group is likely to assume this function. Analogously, Thr1NH_3_^+^ might promote the bivalent reaction mode of epoxyketone inhibitors by protonating the epoxide moiety to create a positively charged trivalent oxygen atom that is subsequently nucleophilically attacked by Thr1NH_2_.

During autolysis the Thr1 N terminus is engaged in a hydroxyoxazolidine ring intermediate (Fig. 3d), which is unstable and short-lived. Breakdown of this tetrahedral transition state releases the Thr1 N terminus that is protonated by aspartic acid 166 via Ser129OH to yield Thr1NH_3_^+^. The residues Ser129 and Asp166 are expected to increase the p*K*_a_ value of Thr1N, thereby favouring its charged state. Consistent with playing an essential role in proton shuttling, the mutation D166A prevents autolysis of the archaeal CP^21^ and the exchange D166N impairs catalytic activity of the yeast CP about 60%. The mutation D166N lowers the p*K*_a_ of Thr1N, which is thus more likely to exist in the uncharged deprotonated state (Thr1NH_2_). This renders the N terminus less suitable to stabilize substrates and to protonate the first cleavage product during catalysis, although it favours its ability to act as a nucleophile. This interpretation agrees with the strongly reduced catalytic activity of the β5-D166N mutant on the one hand, and the ability to react readily with carfilzomib on the other. Hence, the proteasome can be viewed as having a second triad that is essential for efficient proteolysis. While Lys33NH_2_ and Asp17O^δ^ are required to deprotonate the Thr1 hydroxyl side chain, Ser129OH and Asp166OH serve to protonate the N-terminal amine group of Thr1.

In accord with the proposed Thr1–Lys33–Asp17 catalytic triad, crystallographic data on the proteolytically inactive β5-T1C mutant demonstrate that the interaction of Lys33NH_2_ and Cys1 is broken. Consequently, efficient substrate turnover or covalent modification by ligands is prevented. However, owing to Cys being a strong nucleophile, the propeptide can still be cleaved off over time. While only one single turnover is necessary for autolysis, continuous enzymatic activity is required for significant and detectable substrate hydrolysis. Notably, in the Ntn hydrolase penicillin acylase, substitution of the catalytic N-terminal Ser residue by Cys also inactivates the enzyme but still enables precursor processing^33^.

To investigate why the CP specifically employs threonine as its active-site residue, we used a β5-T1S mutant of the yCP and characterized it biochemically and structurally. Activity assays with the β5-T1S mutant revealed reduced turnover of Suc-LLVY-AMC. We also observed slightly lower affinity of the β5-T1S mutant yCP for the Food and Drug Administration-approved proteasome inhibitors bortezomib and carfilzomib. Structural analyses support these findings with the T1S mutant and provide an explanation for the strict use of Thr residues in proteasomes. Thr1 is well anchored in the active site by hydrophobic interactions of its C^γ^ methyl group with Ala46 (C^β^), Lys33 (carbon side chain) and Thr3 (C^γ^). Notably, proteolytically active proteasome subunits from archaea, yeast and mammals, including constitutive, immuno- and thymoproteasome subunits, either encode Thr or Ile at position 3, indicating the importance of the C^γ^ for fixing the position of the nucleophilic Thr1. In contrast to Thr1, the hydroxyl group of Ser1 occupies the position of the Thr1 methyl side chain in the WT enzyme, which requires its reorientation relative to the substrate to allow cleavage (Fig. 4g,h). Notably, in the threonine aspartase Taspase1, mutation of the active-site Thr234 to Ser also places the side chain in the position of the methyl group of Thr234 in the WT, thereby reducing catalytic activity^34^. Similarly, although the serine mutant is active, threonine is more efficient in the context of the proteasome active site. The greater suitability of threonine for the proteasome active site, which has been noted in biochemical as well as in kinetic studies^35^, constitutes a likely reason for the conservation of the Thr1 residue in all proteasomes from bacteria to eukaryotes.

## Methods

### Yeast mutagenesis

Site-directed mutagenesis was performed by standard techniques using oligonucleotides listed in Supplementary Table 2. The *pre2/doa3* (β5) mutant alleles in the centromeric, *TRP1*- or *LEU2*-marked shuttle vectors YCplac22 and pRS315, respectively, were verified by sequencing and subsequently introduced into the yeast strains MHY784 (ref. 1) or YWH20 (ref. 13), which express WT *PRE2* from a *URA3*-marked plasmid. Counter-selection against the *URA3* marker with 5-fluoroorotic acid yielded strains expressing only the mutant forms of β5.

The strain producing a processed β5-T1A variant and the β5 propeptide *in trans* is a derivative of YWH212 (ref. 15). It carries an additional deletion of the *NAT1* gene to avoid *N*-acetylation of Ala1; this strain exhibits extremely slow growth rates and served for crystallographic analysis only. All strains used in this study are listed in Supplementary Table 3.

### Purification of yeast proteasomes

Yeast strains were grown in 18-l cultures at 30 °C in YPD into early stationary phase, and the yCPs were purified according to published procedures^36^. In brief, 120 g yeast cells were solubilized in 150 ml of 50 mM KH_2_PO_4_/K_2_HPO_4_ buffer (pH 7.5) and disrupted with a French press. Cell debris were removed by centrifugation for 30 min at 21,000 r.p.m. (4 °C). The resulting supernatant was filtered and ammonium sulfate (saturated solution) was added to a final concentration of 30% (v/v). This solution was loaded on a Phenyl Sepharose 6 Fast Flow column (GE Healthcare) pre-equilibrated with 1 M ammonium sulfate in 20 mM KH_2_PO_4_/K_2_HPO_4_ (pH 7.5). CPs were eluted by applying a linear gradient from 1 to 0 M ammonium sulfate. Proteasome-containing fractions were pooled and loaded onto a hydroxyapatite column (Bio-Rad) equilibrated with 20 mM KH_2_PO_4_/K_2_HPO_4_ (pH 7.5). Elution of the CPs was achieved by increasing the phosphate buffer concentration from 20 to 500 mM. Anion-exchange chromatogaphy (Resource Q column (GE Healthcare), elution gradient from 0 to 500 mM sodium chloride in 20 mM Tris-HCl (pH 7.5)) and subsequent size-exclusion chromatography (Superose 6 10/300 GL (GE Healthcare), 20 mM Tris-HCl (pH 7.5) and 150 mM NaCl) resulted in pure CPs for crystallization and activity assays.

### Fluorescence-based activity assay

ChT-L (β5) activity of CPs was monitored by fluorescence spectroscopy using the model substrate Suc-LLVY-AMC. Purified yCPs (66 nM in 100 mM Tris-HCl, pH 7.5) were incubated with 300 μM substrate for 1 h at room temperature or 37 °C. The reactions were stopped by diluting samples 1:10 in 20 mM Tris-HCl, pH 7.5. AMC fluorophores released by proteasomal activity were measured in triplicate with a Varian Cary Eclipse Fluorescence Spectrophotometer (Agilent Technologies) at *λ*exc=360 nm and *λ*em=460 nm.

### Inhibition assays

Purified yCPs were mixed with dimethylsulfoxide as a control or serial dilutions of inhibitor and incubated for 45 min at room temperature. A final concentration of yCP of 66 nM was used. After addition of the peptide substrate Suc-LLVY-AMC to a final concentration of 200 μM and incubation for 1 h at room temperature, the reaction was stopped by diluting the samples 1:10 in 20 mM Tris-HCl, pH 7.5. AMC fluorophores released by residual proteasomal activity were measured in triplicate at *λ*exc=360 nm and *λ*em=460 nm. Relative fluorescence units were normalized to the dimethylsulfoxide-treated control. The calculated residual activities were plotted against the logarithm of the applied inhibitor concentration and fitted with GraphPad Prism 5. The IC50 value, the ligand concentration that leads to 50% inhibition of the enzymatic activity, was deduced from the fitted data.

### Crystallization and structure determination

Mutant yCPs were crystallized as previously described for WT 20S proteasomes^36^,^37^. Crystals were grown at 20 °C using the hanging drop vapour diffusion method. Drops contained a 1:1 mixture of protein (40 mg ml^−1^) and reservoir solution (25 mM magnesium acetate, 100 mM 2-(*N*-morpholino)ethanesulfonic acid (MES; pH 6.8) and 9–13% (v/v) 2-methyl-2,4-pentanediol (MPD)). Crystals were cryoprotected by addition of 5 μl cryobuffer (20 mM magnesium acetate, 100 mM MES, pH 6.8, and 30% (v/v) MPD). Inhibitor complex structures were obtained by incubating crystals in 5 μl cryobuffer supplemented with bortezomib or carfilzomib at a final concentration of 1.5 mM for at least 8 h.

Diffraction data were collected at the beamline X06SA at the Paul Scherrer Institute, SLS, Villigen, Switzerland (*λ*=1.0 Å). Evaluation of reflection intensities and data reduction were performed with the programme package XDS^38^. Molecular replacement was carried out with the coordinates of the yeast 20S proteasome (PDB entry code: 5CZ4) by rigid body refinements (REFMAC5; ref. 39). MAIN^40^ and COOT^41^ were used to build models. TLS (Translation/Libration/Screw) refinements finally yielded excellent *R*_work_ and *R*_free_, as well as root mean squared deviation bond and angle values. The coordinates, proven to have good stereochemistry from the Ramachandran plots, were deposited in the RCSB Protein Data Bank (Supplementary Table 1).

The coordinates for the yeast 20S proteasome deposited under the entry code 1RYP do not represent the WT yCP but the double-mutant β5-K33R β1-T1A. At the time of deposition (in 1997), these data were the best available on the yCP. As yCP structure determination has become routine today, and structure refinement procedures have significantly improved, we here provide coordinates for the WT yCP at 2.3 Å resolution (PDB entry code: 5CZ4). Furthermore, the structures of most mutant yCPs described in this work were determined in their apo and ligand-bound states. For mutants with proteolytically inactive β5 subunits, the best crystallographic data obtained are given. For ligands or propeptide segments that were only partially defined in the 2*F*_O_–*F*_C_ electron-density map the occupancy was reduced (for details see Supplementary Table 1).

## Additional information

**Accession codes:** Coordinates and structure factors have been deposited in the Protein Data Bank, www.pdb.org (for PDB entry codes see Supplementary Table 1).

**How to cite this article:** Huber, E. M. *et al*. A unified mechanism for proteolysis and autocatalytic activation in the 20S proteasome. *Nat. Commun.* 7:10900 doi: 10.1038/ncomms10900 (2016).

## Supplementary Material

Supplementary Information Supplementary Figures 1-9, Supplementary Tables 1-3, Supplementary Notes 1-2, Supplementary Methods and Supplementary References.

## Figures and Tables

**Table 1:** Growth phenotypes and status of autolysis and catalysis of mutants.

**Mutant**	**Viability**	**Temperature sensitivity**	Autolysis state of the mutant subunit*	**Activity of the mutant subunit**
WT	++++	+	+	+++
β1-T1A (ref. 13)	++++	+	−	−
β2-T1A (ref. 13)	+++	++	−	−
β1-T1A β2-T1A (ref. 13)	+++	++	−	−
β5-T1A	+/−	++++	−	−
β5-H(-2)A-T1A	+/−	ND	−	−
β5-H(-2)T-T1A	+/−	ND	−	−
β5-H(-2)L-T1A	++	ND	−	−
β5-T1A, pp *trans*, *nat1*Δ	+/−	++++	pp *trans*	−
β5-T1A-K81R	+	++++	−	−
β5-H(-2)A-T1A-K81R	+/−	ND	−	−
β5-H(-2)T-T1A-K81R	+/−	ND	−	−
β5-H(-2)L-T1A-K81R	++	ND	−	−
β5-H(-2)A	++++	++	+	+++
β5-H(-2)K	++++	++	+	+++
β5-H(-2)F	++++	+++	+	++
β5-H(-2)N	++++	+++	+	++
β5pp-β1 (ref. 18)	++++	+	+/−	+/−
β2-T(-2)V	++++	+	−	−
β5-L(-49S)-K33A (ref. 13)	+	++++	−	−
β5-K33A, pp *trans*^13^	+	++++	pp *trans*	+/−
β5-F(-45)S-K33R (ref. 13)	++	++++	+	−
β5-D17N	+/−	++++	ND†	ND†
β5-L(-49)S-D17N	+	++++	+/−	+/−
β5-D17N, pp *trans*	+	++++	pp *trans*	+/−
β5-D166N	++	++++	+	+/−
β5-D166N, pp *trans*	+++	++++	pp *trans*	+/−
β5-T1S	+++	++++	+	++
β5-T1C	++	++++	+	−

ND, not determined.

^*^The autolysis state was assessed by purification and crystallization of the mutant proteasomes.

^†^Purification of this mutant proteasome was not possible.
