# Supplementary material for: A unified mechanism for proteolysis and autocatalytic activation in the 20S proteasome
Source: Nat Commun. 2016 Mar 11;7:10900. doi: 10.1038/ncomms10900 (PMC4792962; doi:10.1038/ncomms10900)
Supplement: Supplementary Information — Supplementary Figures 1-9, Supplementary Tables 1-3, Supplementary Notes 1-2, Supplementary Methods and Supplementary References. [file ncomms10900-s1.pdf]

Supplementary Figures

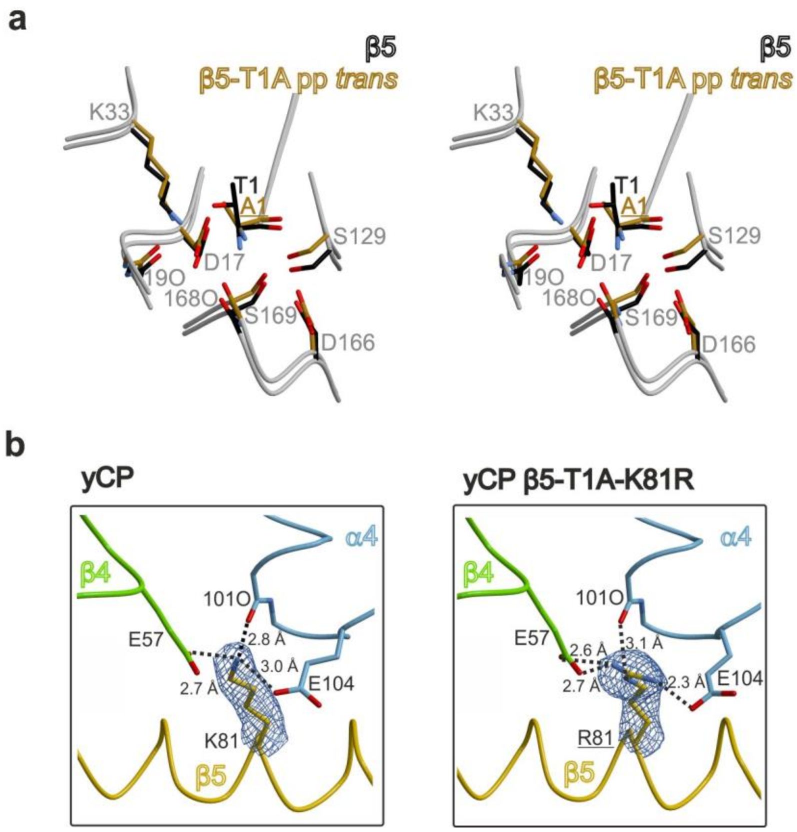

Supplementary Figure 1 Crystallographic analysis of β5-T1A mutants.

(a) Stereo illustration of the WT β5 active site (black) superimposed onto the β5-T1A (pp *trans*) counterpart (brown). The active site architecture is unchanged.

(b) The β5 residue 81 is located at the boundary with subunits α4 and β4. Both Arg81 and Lys81 are well defined in the $2F_O$-F_C electron density map (blue mesh, contoured at 1σ). Distances for hydrogen bonds (black dotted lines) are given. Arg81 is H-bonded to the carbonyl oxygen of Leu101 (α4), to Glu104O$^{\varepsilon}$ (α4) and to Glu57O$^{\varepsilon}$ (β4), similar to Lys81 side chain H-bonding in the WT yCP. However, the guanidinium side chain of Arg81 undergoes an additional interaction with Glu57O$^{\varepsilon}$ (β4). The enhanced inter-subunit interactions resulting from the K81R substitution might weakly improve CP assembly.

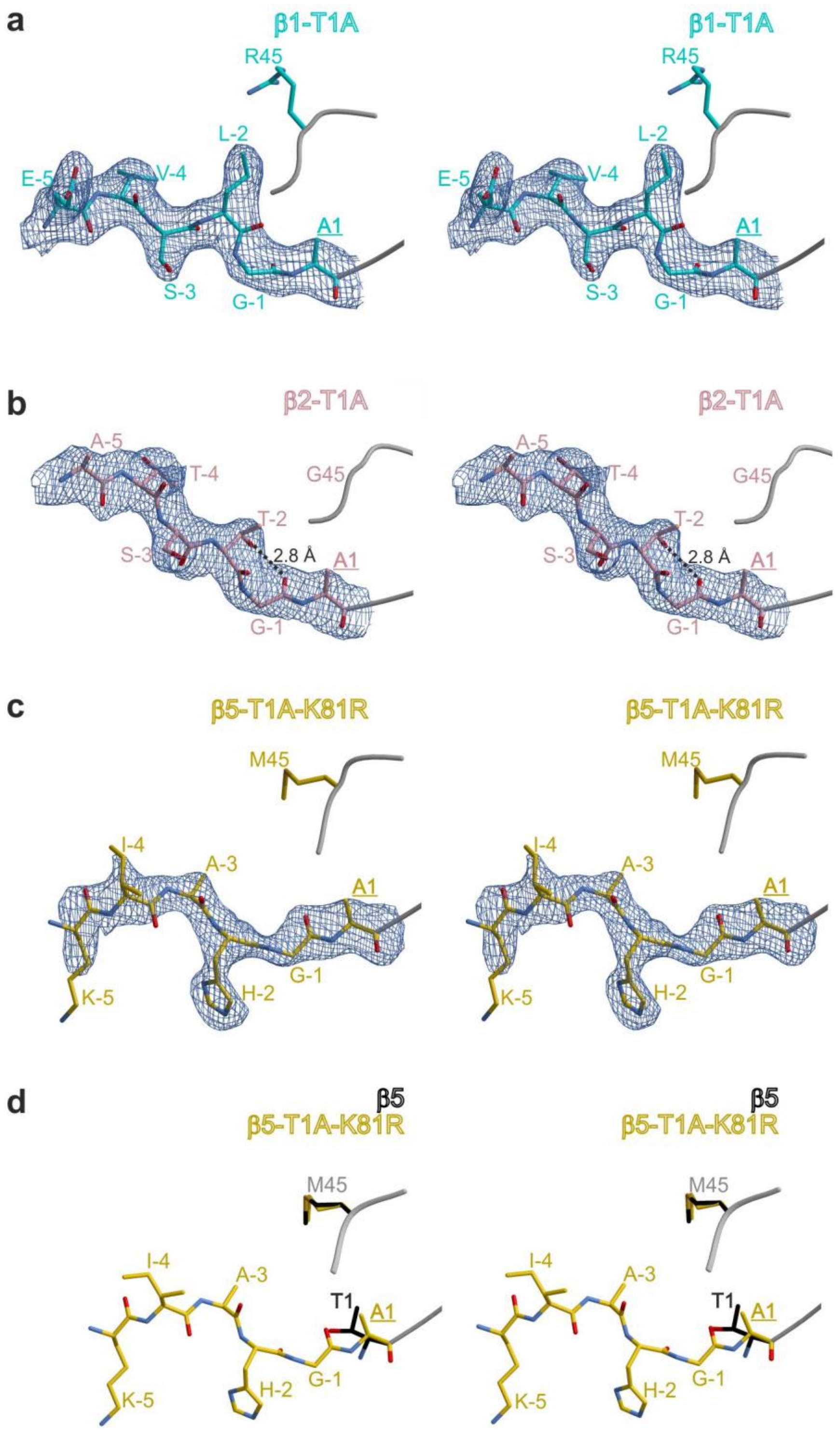

Supplementary Figure 2 Propeptide conformations of T1A mutants.

(a) Stereo illustration of the $2F_O$-F_C electron density map for the β1 propeptide in the β1-T1A mutant proteasome (blue mesh contoured at 1σ). Only the residues (-5)-(-1) of the prosegment are displayed.

(b) Stereo illustration of the $2F_O$-F_C electron density map for the β2 propeptide in the β2-T1A mutant proteasome (blue mesh contoured at 1σ). The hydrogen bond between Thr(-2)OH and Gly(-1)O is indicated by a black dotted line (~2.8 Å).

(c) Stereo illustration of the $2F_O$-F_C electron density map for the β5 propeptide in the β5-T1A-K81R mutant proteasome (blue mesh contoured at 1σ). Notably, His(-2) occupies the S2 instead of the S1 pocket.

(d) Structural superposition of the β5-T1A-K81R (yellow) and the WT β5 active site (black) in stereo. Thr1 is more relaxed compared to Ala1.

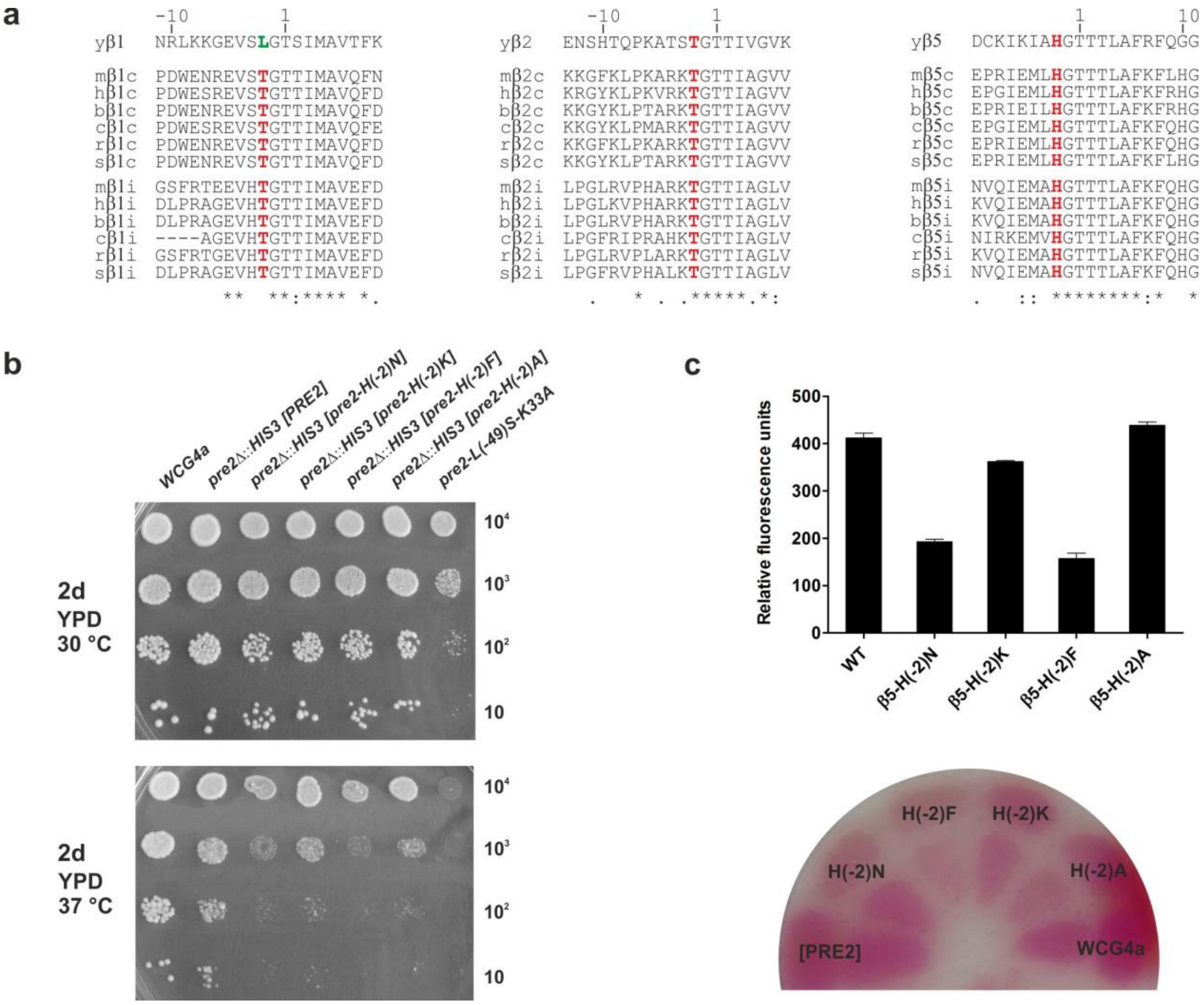

Supplementary Figure 3 The impact of mutations in the β5 propeptide on autolysis and proteasome activity.

(a) Sequence alignment of eukaryotic β1, β2 and β5 propeptide sequences. The (-1) and (-2) amino acids of the propeptide are conserved in both constitutive (c) and immunoproteasome (i) subunits and among most species ((-2) position highlighted in red/green). Leu(-2) in the yeast β1 subunit is an exception (green residue). Abbreviations: m (*Mus musculus*), h (*Homo sapiens*), b (*Bos taurus*), c (*Canis familiaris*), r (*Rattus norvegicus*), s (*Sus scrofa*), y (yeast, *Saccharomyces cerevisiae*).

(b) Serial dilutions and spotting of WT and β5 (*pre2*) mutant yeasts illustrate growth defects after two days of incubation on YPD.

(c) Upper panel: Purified WT and mutant proteasomes were tested for their chymotrypsin-like (ChT-L) activity (β5) using Suc-LLVY-AMC as substrate. Relative fluorescence units were

measured in triplicate after one hour of incubation and are given as mean values. Standard deviations are indicated by black bars. Mutation of His(-2) to Asn or Phe in the β5 propeptide reduces the ChT-L activity of the proteasome, while Lys(-2) and Ala(-2) have no significant impact.

Lower panel: *In situ* test for ChT-L activity of WT and mutant yCPs. Intense pink coloring is indicative of WT-like ChT-L activity towards the substrate Z-GGL-pNA, while pale colonies reflect peptidase defects. All mutants show reduced ChT-L activity compared to WT yeast, suggesting delayed autolysis of the β5 active site.

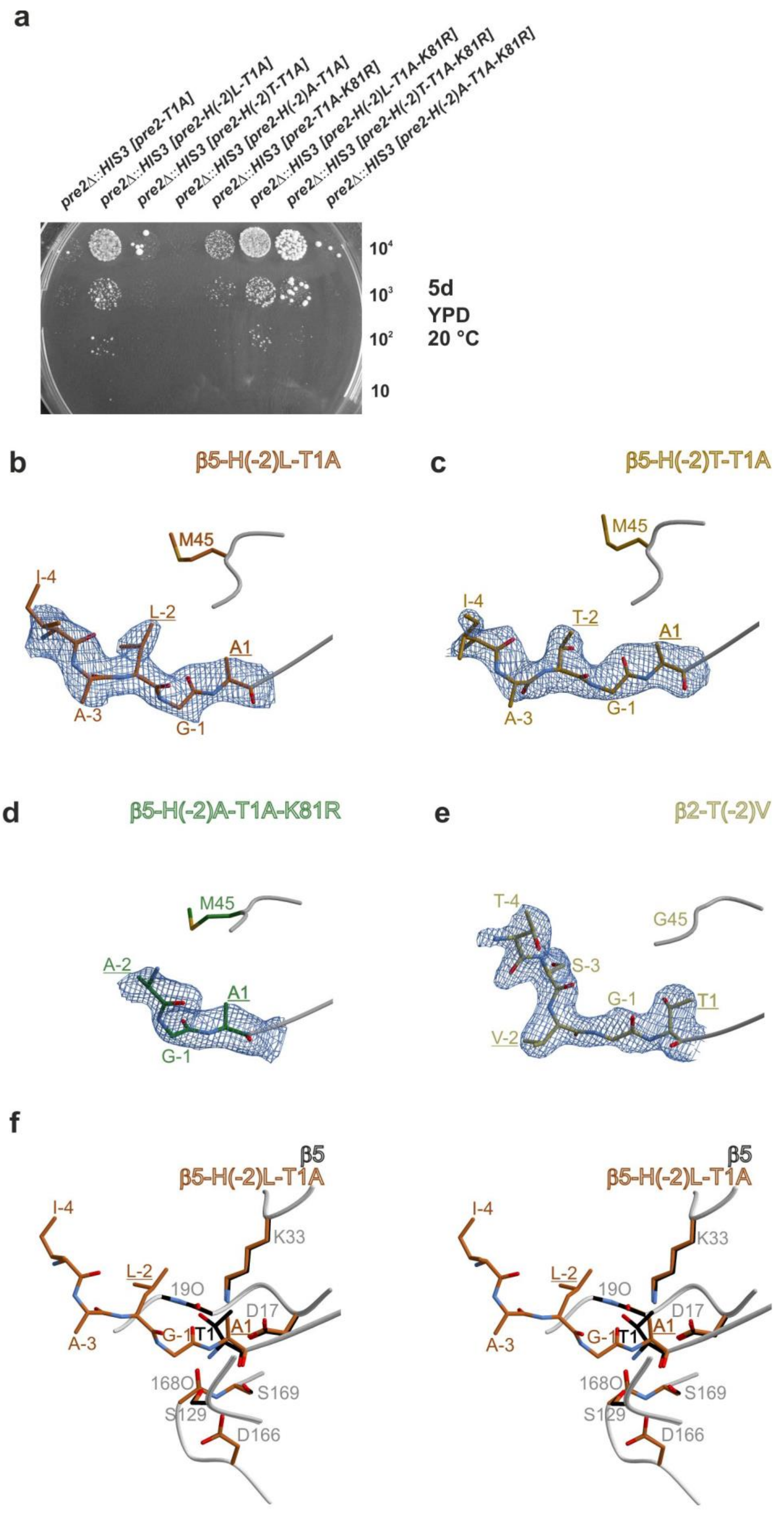
a
pre2Δ::HIS3 [pre2-T1A]
pre2Δ::HIS3 [pre2-H(-2)L-T1A]
pre2Δ::HIS3 [pre2-H(-2)T-T1A]
pre2Δ::HIS3 [pre2-H(-2)A-T1A]
pre2Δ::HIS3 [pre2-T1A-K81R]
pre2Δ::HIS3 [pre2-H(-2)L-T1A-K81R]
pre2Δ::HIS3 [pre2-H(-2)T-T1A-K81R]
pre2Δ::HIS3 [pre2-H(-2)A-T1A-K81R]
10^4
10^3
10^2
10
5d
YPD
20 °C
b
β5-H(-2)L-T1A
M45
I-4
L-2
A1
A-3
G-1
c
β5-H(-2)T-T1A
M45
I-4
T-2
A1
A-3
G-1
d
β5-H(-2)A-T1A-K81R
M45
A-2
A1
G-1
e
β2-T(-2)V
T-4
G45
S-3
G-1
T1
V-2
f
β5
β5-H(-2)L-T1A
I-4
K33
L-2
19O
D17
A-3
G-1
T1
A1
168O
S169
S129
D166

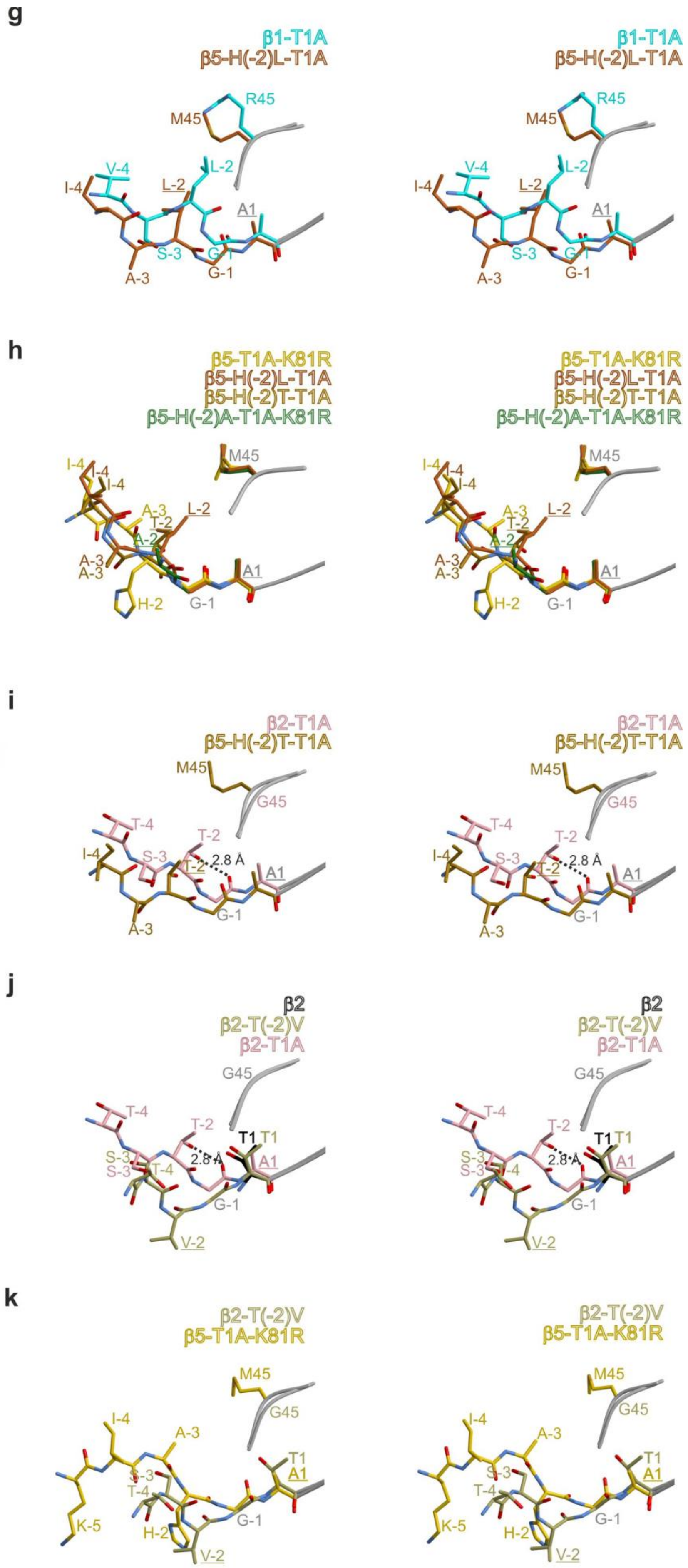
g
β1-T1A
β5-H(-2)L-T1A
R45
M45
V-4
L-2
I-4
L-2
A1
S-3
G-1
A-3
G-1
h
β5-T1A-K81R
β5-H(-2)L-T1A
β5-H(-2)T-T1A
β5-H(-2)A-T1A-K81R
M45
I-4
I-4
I-4
A-3
L-2
T-2
A-2
A-3
A-3
A1
H-2
G-1
i
β2-T1A
β5-H(-2)T-T1A
M45
G45
T-4
T-2
I-4
S-3
T-2
2.8 Å
A1
G-1
A-3
j
β2
β2-T(-2)V
β2-T1A
G45
T-4
T-2
T1
T1
S-3
S-3
T-4
2.8 Å
A1
G-1
V-2
k
β2-T(-2)V
β5-T1A-K81R
M45
G45
I-4
A-3
T1
A1
S-3
T-4
K-5
H-2
G-1
V-2

Supplementary Figure 4 Amino acid exchanges in the β5 propeptide in combination with the active site mutant T1A.

(a) Serial dilutions and spotting of β5 (*pre2*) mutant yeasts illustrate growth defects after five days of incubation on YPD at 20 °C. Note that among the small colonies of *pre2*-T1A, *pre2*-H(-2)T-T1A as well as *pre2*-H(-2)T-T1A-K81R and *pre2*-H(-2)A-T1A-K81R singular large colonies presumably originate from spontaneously acquired suppressor mutations. The mutant *pre2*-H(-2)L-T1A(-K81R) grows the best, when ignoring suppressor colonies.

(b) Illustration of the $2F_O$-F_C electron density map for the β5 propeptide in the β5-H(-2)L-T1A mutant proteasome (blue mesh contoured at 1σ).

(c) Illustration of the $2F_O$-F_C electron density map for the β5 propeptide in the β5-H(-2)T-T1A mutant proteasome (blue mesh contoured at 1σ).

(d) Illustration of the $2F_O$-F_C electron density map for the β5 propeptide in the β5-H(-2)A-T1A-K81R mutant proteasome (blue mesh contoured at 1σ).

(e) Illustration of the $2F_O$-F_C electron density map for the β2 propeptide of the β2-T(-2)V mutant proteasome (blue mesh, contoured at 1σ). The mutation T(-2)V disrupts the hydrogen bond between Thr(-2) and Gly(-1)O (panel j), thereby leading to a flip of Gly(-1) and to the inhibition of autolysis.

(f) Stereo view of the structural superposition of the β5 active site of the β5-H(-2)L-T1A mutant and the WT yeast proteasome. The mutant subunit is not matured and thus carries its propeptide. All active site residues are in place except for Thr1 which has been mutated to Ala.

(g-j) Stereo views of Fig. 2a-d.

(k) Structural superposition of the β2-T(-2)V and the β5-T1A-K81R propeptide illustrates the different conformations of Gly(-1)O.

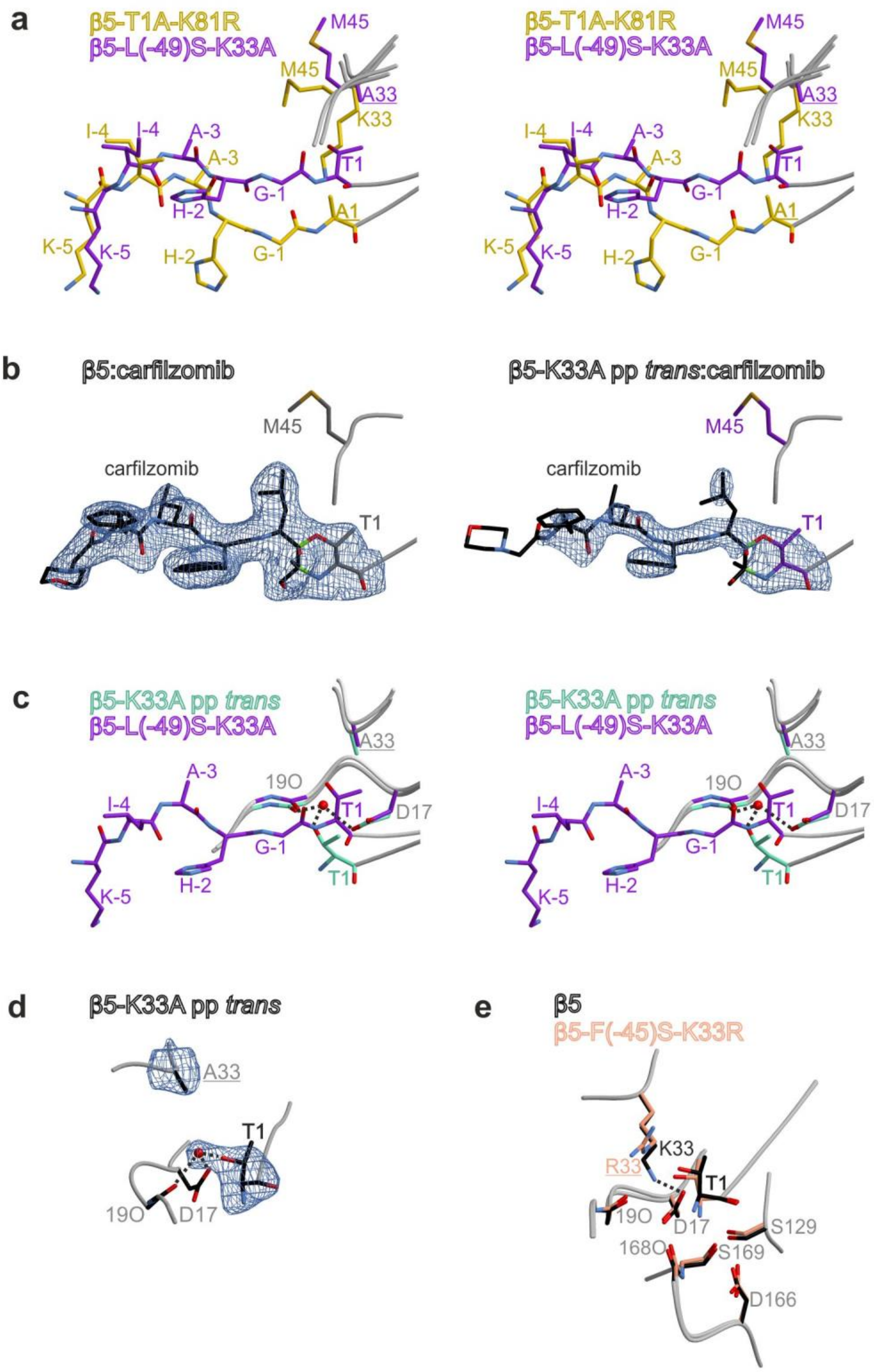

Supplementary Figure 5 Mutations in the active site of the proteasome and their impact on autolysis and the active site.

(a) Stereo illustration of the β5-T1A-K81R subunit superimposed onto the β5-K33A mutant active site. Only the residues (-5)-(-1) of the β5-K33A propeptide are displayed. In both structures His-2 does not occupy the S1 pocket formed by Met45. Notably, the mutation

K33A creates additional space around the active site that is filled by Thr1 and the propeptide, thereby causing Met45 to rotate.

(b) Illustration of the $2F_O$-F_C electron density (blue mesh, contoured at 1σ) for carfilzomib bound to the WT β5 active site (left) and to the β5-K33A pp *trans* mutant subunit (right). Carfilzomib is poorly defined at the β5-K33A pp *trans* mutant active site (2.7 Å resolution) compared to the WT structure (PDB ID: 4QW4[1]; 2.8 Å resolution), indicating that the reactivity of Thr1 in the mutant subunit is reduced.

(c) Stereo views of the structural superposition of the active sites of the β5-K33A mutant proteasomes with the propeptide expressed in *cis* (purple) or *trans* (turquoise). The singular water molecule only found in the β5-K33A pp *trans* mutant is depicted in red, whereas the associated hydrogen bonding network is indicated by black dashed lines.

(d) In the active site of the β5-K33A pp *trans* mutant proteasome a defined water molecule (red sphere) is bound to Thr1OH, Arg19O and Asp17O^{δ}. Hydrogen bonds are depicted by black dotted lines. This water molecule may enable residual catalytic activity of this mutant by activating Thr1O^{γ}. The $2F_O$-F_C electron density map for Ala33, Thr1 and the peculiar water molecule is provided as a blue mesh contoured at 1σ.

(e) The WT proteasome subunit β5 (black) is superimposed onto the β5-K33R mutant subunit (salmon; PDB ID: 1RYP[2]). Mutation of Lys33 to Arg allows propeptide processing based on the structural data, but it strongly retards β5 maturation *in vivo*[3] and is incompatible with proteolytic activity in the mature proteasome. Compared to lysine, arginine is elongated and therefore sterically more demanding. To prevent clashes with its protein surroundings, the guanidinium group of Arg33 is tilted. This conformational change disrupts the interaction with Asp17 and inactivates the proteasome.

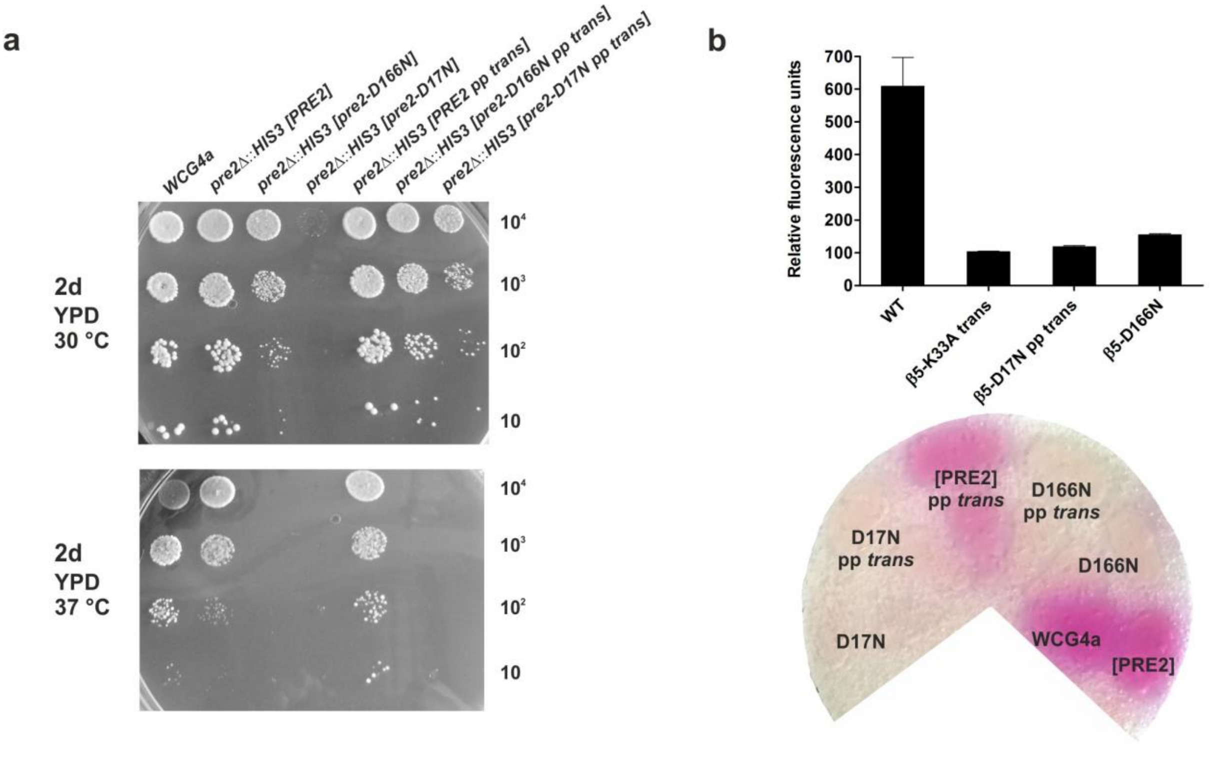

Supplementary Figure 6 Active site mutants of the proteasome and their effect on yeast growth and proteasome activity.

(a) Serial dilutions and spotting of WT and β5 (*pre2*) mutant yeasts illustrate the growth defects of the D17N and D166N mutants after two days of incubation on YPD.

(b) Upper panel: Purified WT and mutant proteasomes were tested for their ChT-L activity (β5) using Suc-LLVY-AMC as substrate. Relative fluorescence units were measured in triplicate after one hour of incubation and are given as mean values. Standard deviations are indicated (black bars). Both the β5-D17N pp *trans* and β5-D166N mutants are strongly reduced in their ChT-L activity compared to WT yCP.

Lower panel: *In situ* test for ChT-L activity of WT and mutant yCPs. Intense pink coloring is indicative of WT-like ChT-L activity towards the substrate Z-GGL-pNA, while pale colonies reflect peptidase defects.

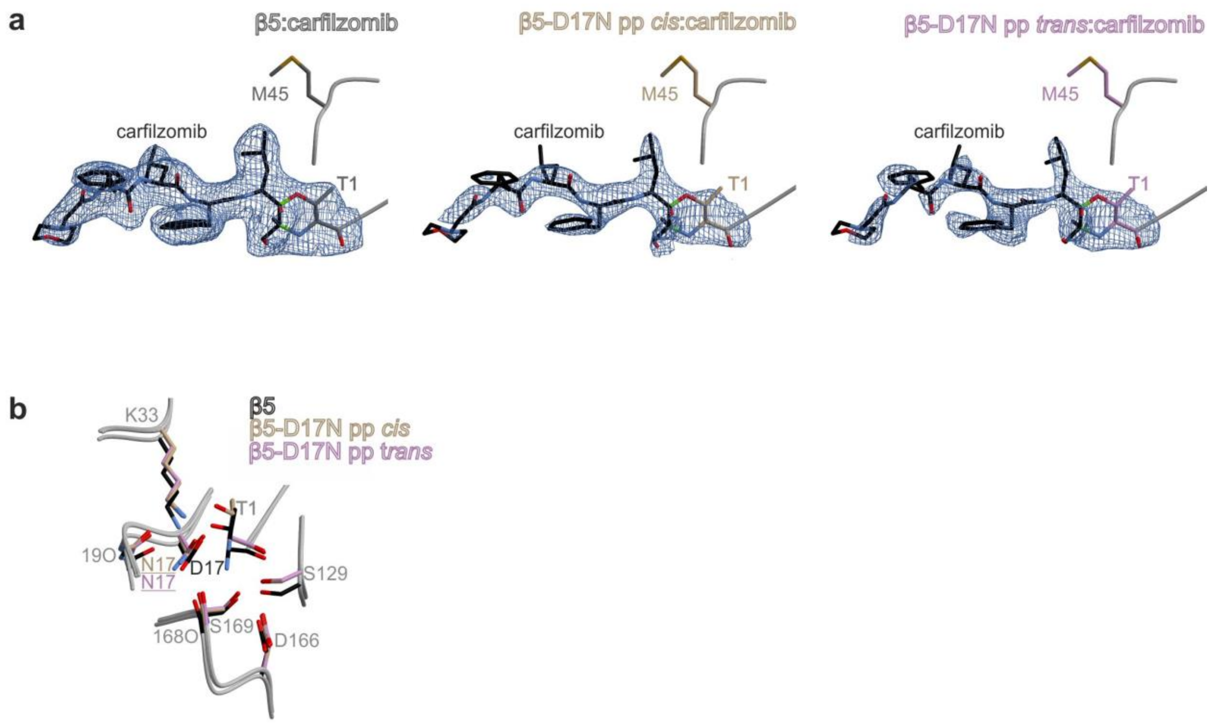

Supplementary Figure 7 Structural consequences of the β5-D17N mutation.

(a) Illustration of the $2F_O$-F_C electron density (blue mesh, contoured at 1σ) for carfilzomib bound to the WT β5 active site (left) and to the β5-D17N mutant subunit with the propeptide expressed in *cis* (middle) as well as in *trans* (right). Carfilzomib is bound to the WT β5 subunit at full occupancy (PDB ID: 4QW4[1]; 2.8 Å resolution) while both mutant active sites show only residual electron density for the ligand (pp *cis*: 2.7 Å resolution; pp *trans*: 2.8 Å resolution).

(b) Superposition of the β5 WT and the β5-D17N mutant active sites illustrates their structural similarity.

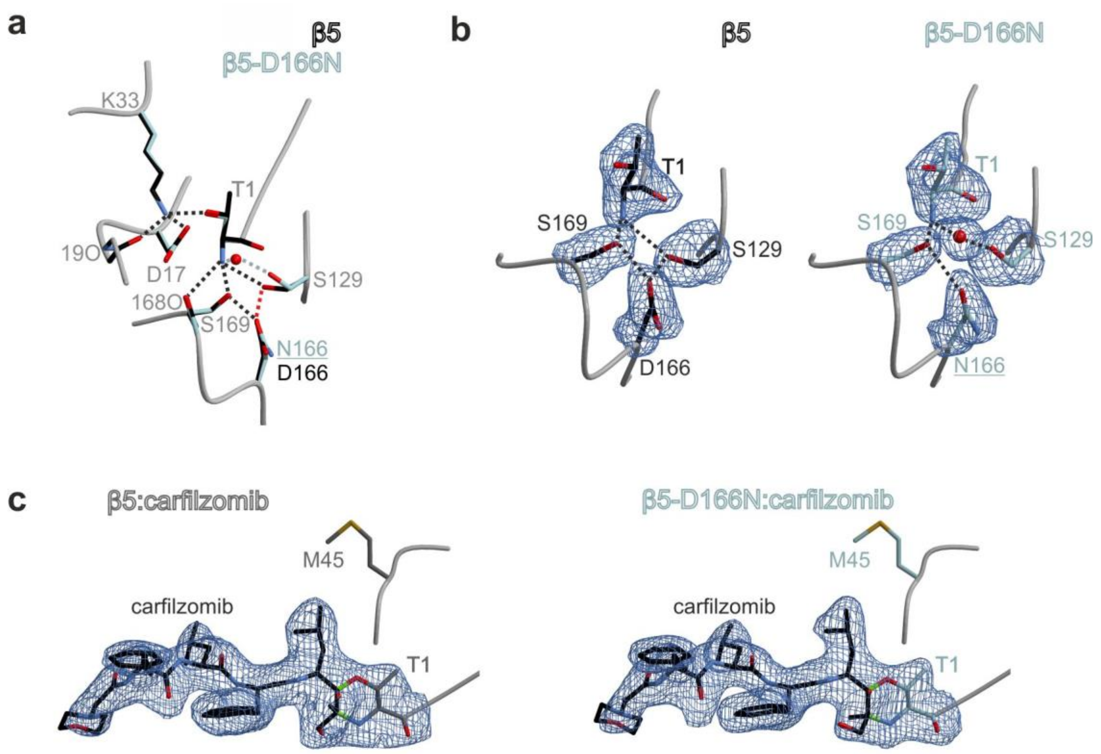

Supplementary Figure 8 Structural consequences of the β5-D166N mutation.

(a) Superposition of the β5 WT (black) and the β5-D166N mutant (light blue) active sites highlights their similarity. In the mutant however, Ser129 is rotated and the hydrogen bond to Asp166 is broken (red dotted line). Instead a water molecule bridges Thr1NH_3^+ and Ser129OH (gray dashed lines). Presumably, this altered H-bonding pattern decreases the catalytic activity of the β5-D166N mutant. The hydrogen bonds involving Ser169OH are unchanged in the mutant and may enable autolysis as well as residual substrate turnover.

(b) Illustration of the $2F_O$-F_C electron density map (blue mesh, contoured at 1 σ) for the WT and D166N mutant β5 active sites. In the WT, Ser129OH hydrogen bridges to Asp166O^δ and Thr1NH_3^+, whereas in the mutant, Ser129OH interacts with Thr1NH_3^+ via a water molecule.

(c) Illustration of the $2F_O$-F_C electron density (blue mesh, contoured at 1σ) for carfilzomib bound to the WT β5 active site (left) and to the β5-D166N mutant subunit (right). Carfilzomib is equally well defined in the WT (PDB ID: 4QW4[1]; 2.8 Å resolution) and mutant active sites (2.5 Å resolution).

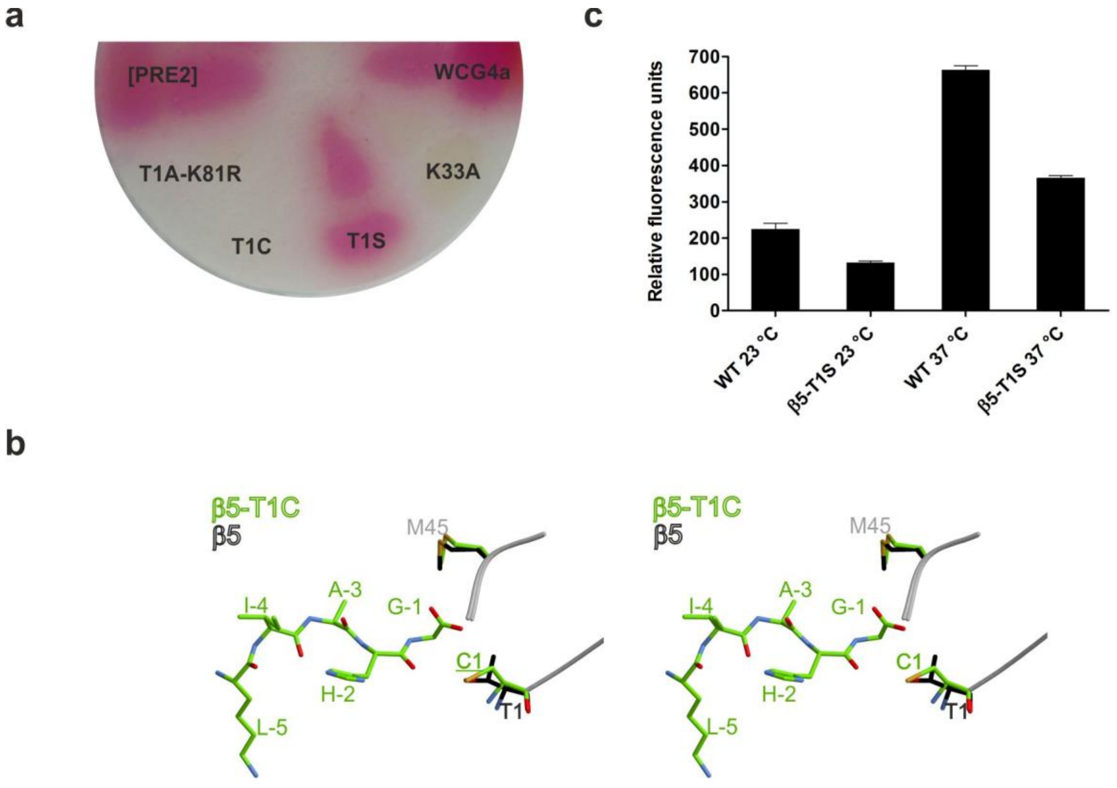

Supplementary Figure 9 The active site nucleophile: threonine versus serine and cysteine.

(a) *In situ* test for ChT-L activity of WT and mutant yCPs. Intense pink coloring is indicative of high ChT-L activity towards the substrate Z-GGL-pNA, while pale coloring reflects strongly reduced ChT-L activity.

(b) Stereo illustration of the β5-T1C active site superimposed onto the WT counterpart. This figure is provided to allow stereo viewing of the different orientations of Cys1 and Thr1 (related to the non-stereo views in Fig. 4c-e).

(c) Purified WT and mutant proteasomes were tested for their ChT-L activity using Suc-LLVY-AMC as substrate at two different temperatures. Relative fluorescence units were measured in triplicate after one hour of incubation and are given as mean values. Standard deviations are indicated (black bars). The catalytic activity of the β5-T1S mutant is reduced by ~41% at 23 °C and by 45 % at 37 °C, respectively compared to WT proteasomes.

Supplementary Tables

Supplementary Table 1 Data collection and refinement statistics

	WT yCP	*yCPβ1-T1A: carfilzomib*	*yCPβ2-T1A*	*yCPβ1-T1A-β2-T1A: carfilzomib*	*yCPβ5-T1A pp trans: SylA*	*yCPβ5-T1A-K81R: bortezomib*
**Data collection**						
Space group	$P2_1$	$P2_1$	$P2_1$	$P2_1$	$P2_1$	$P2_1$
Cell dimensions						
a, *b*, *c* (Å)	137.0, 300.9, 145.8	136.8, 300.4, 145.3	135.0, 301.7, 144.0	136.8, 301.2, 145.9	135.0, 301.4, 145.7	136.1, 301.7, 145.5
α, β, γ (°)	90.0, 113.1, 90.0	90.0, 113.1, 90.0	90.0, 113.1, 90.0	90.0, 113.3, 90.0	90.0, 113.3, 90.0	90.0, 113.4, 90.0
Resolution (Å)[a]	30-2.3 (2.4-2.3)	30-2.8 (2.9-2.8)	30-2.7 (2.8-2.7)	30-2.9 (3.0-2.9)	30-2.7 (2.8-2.7)	30-2.5 (2.6-2.5)
R_{merge} (%)	6.1 (47.9)	6.7 (45.7)	7.7 (52.8)	8.0 (49.8)	7.8 (51.3)	5.6 (45.3)
$I / \sigma I$	13.1 (2.0)	15.1 (2.7)	12.0 (2.6)	11.7 (2.8)	9.8 (2.2)	17.1 (3.5)
Completeness (%)	98.7 (92.7)	97.9 (97.2)	94.7 (98.0)	97.1 (98.9)	97.4 (99.4)	99.6 (99.6)
Redundancy	3.3 (2.3)	3.1 (2.9)	3.1 (3.1)	3.1 (3.2)	2.9 (3.0)	3.8 (3.7)
**Refinement**						
Resolution (Å)	15-2.3	15-2.8	15-2.7	15-2.9	15-2.7	15-2.5
No. reflections	449280	245669	260437	220501	270355	350199
R_{work} / R_{free} (%)	21.1/22.8	20.1/22.2	20.6/23.1	18.6/21.0	19.4/22.6	19.7/21.5
No. atoms						
Protein	49373	49449	49378	49716	49372	49448
Ligand/ion	11	224	10	130	81	127
Water	1522	321	605	228	448	1117
B-factors						
Protein	56.4	66.6	56.3	66.1	63.9	57.3
Ligand/ion	42.9	60.5	47.0	57.4	54.3	44.4
Water	47.5	44.3	43.5	42.6	47.5	43.5
R.m.s. deviations						
Bond lengths (Å)	0.005	0.005	0.004	0.004	0.004	0.004
Bond angles (°)	0.888	0.861	0.859	0.858	0.859	0.836
Ramachandran Plot (%)[b]	97.9/1.8/0.3	97.6/2.2/0.2	97.8/1.9/0.3	97.6/2.1/0.3	97.8/1.9/0.3	97.9/1.8/0.3
PDB accession code	5CZ4	5CZ5	5FG7	5FGI	5CZ6	5CZ7

[a] Values in parentheses are for highest-resolution shell. Data were collected from a single crystal.
[b] Number of residues in favored region / allowed region / outlier region.

	yCPβ5-H(-2)L-T1A: carfilzomib	*yCPβ5-H(-2)T-T1A: carfilzomib*	*yCPβ5-H(-2)A-T1A-K81R: carfilzomib*	*yCP:β2T(-2)V*	*yCPβ5-L(-49)S-K33A: carfilzomib*	*yCPβ5-K33A pp trans*
**Data collection**						
Space group	$P2_1$	$P2_1$	$P2_1$	$P2_1$	$P2_1$	$P2_1$
Cell dimensions						
a, *b*, *c* (Å)	136.8, 300.9, 145.8	136.2, 301.6, 147.8	136.1, 301.0, 144.9	135.6, 301.5, 145.1	136.4, 299.3, 144.5	135.4, 300.5, 144.4
α, β, γ (°)	90.0, 113.1, 90.0	90.0, 113.2, 90.0	90.0, 113.1, 90.0	90.0, 113.4, 90.0	90.0, 113.1, 90.0	90.0, 113.1, 90.0
Resolution (Å)[a]	30-2.8 (2.9-2.8)	30-2.6 (2.7-2.6)	30-2.6 (2.7-2.6)	30-2.6 (2.7-2.6)	30-2.8 (2.9-2.8)	30-2.7 (2.8-2.7)
R_{merge} (%)	8.7 (45.2)	7.4 (53.3)	7.0 (45.5)	9.3 (45.7)	7.0 (53.6)	9.7 (51.4)
$I / \sigma I$	10.5 (3.3)	12.3 (2.9)	12.7 (3.1)	8.9 (2.0)	11.4 (2.3)	9.4 (2.6)
Completeness (%)	98.2 (96.6)	98.5 (99.0)	98.6 (99.2)	97.1(93.5)	96.2 (97.5)	98.5 (97.8)
Redundancy	3.8 (3.5)	3.9 (3.8)	3.8 (3.8)	3.3 (3.2)	3.1 (2.9)	3.9 (3.8)
**Refinement**						
Resolution (Å)	15-2.8	15-2.6	15-2.6	15-2.6	15-2.8	15-2.7
No. reflections	247741	309276	306812	301664	238627	271226
R_{work} / R_{free} (%)	18.2/20.8	19.0/21.4	19.2/21.4	20.9/23.0	19.1/21.5	18.8 /21.4
No. atoms						
Protein	49363	49403	49329	49425	49430	49359
Ligand/ion	228	227	229	9	222	10
Water	282	374	608	725	211	512
B-factors						
Protein	67.7[‖]	66.2[‖]	63.2[†]	57.0	77.7	63.7
Ligand/ion	68.8	67.9	63.7	48.8	76.3	57.9
Water	49.2	51.3	48.5	41.8	52.6	46.3
R.m.s. deviations						
Bond lengths (Å)	0.004	0.004	0.005	0.004	0.004	0.005
Bond angles (°)	0.883	0.866	0.906	0.869	0.852	0.900
Ramachandran Plot (%)[b]	97.6/2.2/0.2	97.6/2.2/0.2	97.7/2.1/0.2	97.8/1.9/0.3	97.6/2.2/0.2	97.8/1.9/0.3
PDB accession code	5FGD	5FGE	5FGF	5FG9	5CZ8	5FGA

[a] Values in parentheses are for highest-resolution shell. Data were collected from a single crystal.
[b] Number of residues in favored region / allowed region / outlier region.
[‖] The occupancy of parts of the β5 propeptide (in chain K) has been set to 0.75.
[†] The occupancy of parts of the β5 propeptides has been set to 0.8.

	yCPβ5-K33A pp trans: MG132	*yCPβ5-K33A pp trans: carfilzomib*	*yCPβ5-(L-49S)-D17N:carfilzomib*	*yCPβ5-D17N pp trans: carfilzomib*	*yCPβ5-D166N*	*yCPβ5-D166N: carfilzomib*
**Data collection**						
Space group	$P2_1$	$P2_1$	$P2_1$	$P2_1$	$P2_1$	$P2_1$
Cell dimensions						
a, *b*, *c* (Å)	136.9, 299.9, 146.0	136.3, 300.8, 145.0	134.3, 300.5, 144.3	136.8, 300.4, 145.3	135.0, 301.4, 145.7	136.1, 301.7, 145.5
α, β, γ (°)	90.0, 113.1, 90.0	90.0, 113.2, 90.0	90.0, 112.7, 90.0	90.0, 113.1, 90.0	90.0, 113.3, 90.0	90.0, 113.4, 90.0
Resolution (Å)[a]	30-2.8 (2.9-2.8)	30-2.7 (2.8-2.7)	30-2.7 (2.8-2.7)	30-2.8 (2.9-2.8)	30-2.5 (2.6-2.5)	30-2.5 (2.6-2.5)
R_{merge} (%)	7.9 (48.6)	7.2 (47.4)	7.2 (51.9)	8.3 (53.3)	5.9 (53.2)	6.6 (49.4)
$I / \sigma I$	11.7 (3.0)	12.0 (2.9)	12.4 (3.0)	14.6 (2.9)	16.7 (2.7)	13.9 (3.2)
Completeness (%)	97.3 (98.0)	98.4 (98.2)	97.2 (98.5)	94.6 (96.8)	97.4 (98.3)	96.3 (95.4)
Redundancy	3.9 (3.8)	3.8 (3.8)	3.2 (3.2)	3.1 (3.2)	3.4 (3.4)	3.2 (3.1)
**Refinement**						
Resolution (Å)	15-2.8	15-2.7	15-2.7	15-2.9	15-2.5	15-2.5
No. reflections	245092	174019	266158	212352	337017	333089
R_{work} / R_{free} (%)	18.3/20.7	18.7/21.2	18.9/20.8	19.9/22.3	20.6/22.4	21.6/23.7
No. atoms						
Protein	49375	49625	49335	49320	49333	49303
Ligand/ion	216	303	348	327	10	327
Water	261	314	409	340	1198	1100
B-factors						
Protein	71.0	66.6	63.2	58.6	55.4	56.0
Ligand/ion	119.0	70.0[‡]	67.4[*]	62.3[*]	43.1	54.3
Water	53.2	50.2	47.4	38.5	42.2	40.3
R.m.s. deviations						
Bond lengths (Å)	0.004	0.005	0.004	0.005	0.004	0.004
Bond angles (°)	0.855	0.899	0.888	0.868	0.849	0.869
Ramachandran Plot (%)[b]	97.8/1.9/0.3	97.6/2.1/0.2	97.5/2.3/0.2	97.6/2.2/0.2	97.9/1.8/0.3	97.7/2.1/0.2
PDB accession code	5FGH	5FHS	5FGG	5CZ9	5CZA	5D0S

[a] Values in parentheses are for highest-resolution shell. Data were collected from a single crystal.
[b] Number of residues in favored region / allowed region / outlier region.
* The occupancy of the ligands bound to the β5 subunits has been set to 0.4.
‡ The ligand is bound only to one of the two β5 subunits (chain K) and its occupancy has been set to 0.5.

	yCPβ5-D166N: MG132	*yCPβ5-T1C: carfilzomib*	*yCPβ5-T1S*	*yCPβ5-T1S: bortezomib*	*yCPβ5-T1S: carfilzomib*
**Data collection**					
Space group	$P2_1$	$P2_1$	$P2_1$	$P2_1$	$P2_1$
Cell dimensions					
a, *b*, *c* (Å)	136.9, 300.1, 145.1	135.2, 299.9, 144.9	136.4, 301.1, 145.4	136.8, 300.2, 145.3	135.3, 300.7, 144.4
α, β, γ (°)	90.0, 113.3, 90.0	90.0, 112.6, 90.0	90.0, 113.0, 90.0	90.0, 113.1, 90.0	90.0, 112.9, 90.0
Resolution (Å)[a]	30-2.6 (2.7-2.6)	30-2.9 (3.0-2.9)	30-2.5 (2.6-2.5)	30-2.6 (2.7-2.6)	30-2.9 (3.0-2.9)
R_{merge} (%)	6.3 (48.8)	8.0 (44.7)	6.6 (57.8)	6.3 (57.5)	7.6 (43.2)
$I / \sigma I$	13.2 (2.5)	10.2 (2.6)	11.1 (2.3)	14.5 (2.7)	11.0 (2.2)
Completeness (%)	98.0 (99.1)	97.6 (99.1)	95.7 (98.0)	97.5 (98.7)	96.0 (97.3)
Redundancy	3.1 (3.1)	3.0 (3.0)	2.9 (3.1)	3.1 (3.2)	2.8 (2.8)
**Refinement**					
Resolution (Å)	15-2.6	15-2.9	15-2.5	15-2.6	15-2.9
No. reflections	306144	217817	337637	305250	213753
R_{work} / R_{free} (%)	19.5/21.8	19.3/22.0	20.1/21.9	20.4/22.4	17.8/20.7
No. atoms					
Protein	49351	49346	49206	49230	49945
Ligand/ion	151	222	11	180	312
Water	598	288	470	561	314
B-factors					
Protein	65.1	69.8	66.1	65.1	69.9
Ligand/ion	95.4	64.4	56.9	50.6	66.8
Water	46.8	40.4	44.7	47.2	46.1
R.m.s. deviations					
Bond lengths (Å)	0.004	0.004	0.004	0.004	0.005
Bond angles (°)	0.878	0.855	0.862	0.857	0.863
Ramachandran Plot (%)[b]	97.8/1.9/0.3	97.6/2.2/0.2	97.9/1.8/0.3	97.7/2.0/0.3	97.7/2.1/0.2
PDB accession code	5D0T	5D0V	5D0W	5D0X	5D0Z

[a]Values in parentheses are for highest-resolution shell. Data were collected from a single crystal.
[b]Number of residues in favored region / allowed region / outlier region.

Supplementary Table 2
Primer sequences used in this study.

Oligonucleotide	Sequence 5' → 3'
Pre2-T1C_for	GCACATGGTTGTACAACCTTAGCATTTAG
Pre2-T1C_rev	CTAAGGTTGTACAACCATGTGCGATCTTG
Pre2-H(-2)A_for	GACTGTAAAATCAAGATCGCAGCCGGTACTACAACCTTAGC
Pre2-H(-2)A_rev	GCTAAGGTTGTAGTACCGGCTGCGATCTTGATTTTACAGTC
Pre2-H(-2)F_for	CCCAGACTGTAAAATCAAGATCGCATTCGGTACTACAACCTTAGC
Pre2-H(-2)F_rev	GCTAAGGTTGTAGTACCGAATGCGATCTTGATTTTACAGTCTGGG
Pre2-H(-2)K_for	CAGACTGTAAAATCAAGATCGCAAAGGGTACTACAACCTTAGC
Pre2-H(-2)K_rev	GCTAAGGTTGTAGTACCCTTTGCGATCTTGATTTTACAGTCTG
Pre2-H(-2)N_for	CAAGATCGCAAATGGTACTACAACC
Pre2-H(-2)N_rev	GTAGTACCATTTGCGATCTTGATTTTAC
Pre2-D17N_for	GTGGCAGTAAATTCTCGTGCCACTGCCG
Pre2-D17N_rev	GCACGAGAATTTACTGCCACAATAATACC
Pre2-D166N_for	GCTGCCCATAGAAATGCTTACTCTGGTGG
Pre2-D166N_rev	CCAGAGTAAGCATTTCTATGGGCAGCAGC
Pre2-H(-2)L-T1A_for	GTAAAATCAAGATCGCACTTGGTGCTACAACCTTAGC
Pre2-H(-2)L-T1A_rev	GCTAAGGTTGTAGCACCAAGTGCGATCTTGATTTTAC
Pre2-H(-2)A-T1A_for	CCCAGACTGTAAAATCAAGATCGCAGCTGGTGCTACAACC
Pre2-H(-2)A-T1A_rev	GGTTGTAGCACCAGCTGCGATCTTGATTTTACAGTCTGGG
Pre2-H(-2)T-T1A_for	CCCAGACTGTAAAATCAAGATCGCAACTGGTGCTACAACC
Pre2-H(-2)T-T1A_rev	GGTTGTAGCACCAGTTGCGATCTTGATTTTACAGTCTGGG
Pup1-T(-2)V_for	GCAACTTCCGTGGGTACCACCATTGTAG
Pup1-T(-2)V_rev	GGTGGTACCCACGGAAGTTGCCTTAGG

Supplementary Table 3
Yeast strains used in this study

Strain	Genotype	Source
MHY784	*MATα his3-Δ200 leu2-3,112 ura3-52 lys2-801 trp1-1 pre2-Δ1::HIS3* [YCp50-PRE2]	Chen and Hochstrasser, 1996[4]
YWH227	*MATα his3-Δ200 leu2-3,112 ura3-52 lys2-801 trp1-1 pre2-Δ1::HIS3* [YCplac22-pre2-H(−2)A]	this study
YWH228	*MATα his3-Δ200 leu2-3,112 ura3-52 lys2-801 trp1-1 pre2-Δ1::HIS3* [YCplac22-pre2-H(−2)K]	this study
YWH229	*MATα his3-Δ200 leu2-3,112 ura3-52 lys2-801 trp1-1 pre2-Δ1::HIS3* [YCplac22-pre2-H(−2)F]	this study
YWH217	*MATα his3-Δ200 leu2-3,112 ura3-52 lys2-801 trp1-1 pre2-Δ1::HIS3* [YCplac22-pre2-H(−2)N]	this study
YWH216	*MATα his3-Δ200 leu2-3,112 ura3-52 lys2-801 trp1-1 pre2-Δ1::HIS3* [YCplac22-pre2-T1C]	this study
YWH1003	*MATα his3-Δ200 leu2-3,112 ura3-52 lys2-801 trp1-1 pre2-Δ1::HIS3* [YCplac22-pre2-T1A-K81R]	as MHY1003 in Chen and Hochstrasser, 1996[4], but without His-tag
YWH2215	*MATα his3-Δ200 leu2-3,112 ura3-52 lys2-801 trp1-1 pre2-Δ1::HIS3* [pRS315-pre2-T1S]	this study, plasmid same as p15-E2T76S in Heinemeyer et al., 1997[3]
MHY1132	*MATα his3-Δ200 leu2-3,112 ura3-52 lys2-801 trp1-1 pre3Δ::HIS3* [pRS315-PRE2LS-pre3ΔLS]	Li et al., 2015[5]
WCG4a	*MAT***a** *his3-11,15 leu2-3,112 ura3*	Heinemeyer et al., 1993[6]
YWH23	*MAT***a** *his3-11,15 leu2-3,112 ura3 pre2-L(−49)S-K33A*	Heinemeyer et al., 1997[3]
YWH20	*MAT***a** *his3-11,15 leu2-3,112 ura3 pre2Δ::HIS3* [pRS316-PRE2]	Heinemeyer et al., 1997[3]
YWH218	*MAT***a** *his3-11,15 leu2-3,112 ura3 pre2Δ::HIS3* [pRS315-pre2-D17N]	this study
YWH233	*MAT***a** *his3-11,15 leu2-3,112 ura3 pre2Δ::HIS3* [pRS315-pre2-L(-49)S-D17N]	this study
YWH219	*MAT***a** *his3-11,15 leu2-3,112 ura3 pre2Δ::HIS3* [pRS315-pre2-D166N]	this study
YUS1	*MAT***a** *his3-11,15 leu2-3,112 ura3 pre3-T1A*	Heinemeyer et al., 1997[3]
YUS4	*MAT***a** *his3-11,15 leu2-3,112 ura3 pup-T1A*	Heinemeyer et al., 1997[3]
YUS5	*MAT***a** *his3-11,15 leu2-3,112 ura3 pre3-T1A pup1-T1A*	Heinemeyer et al., 1997[3]
YWH230	*MAT***a** *his3-11,15 leu2-3,112 ura3 pup1Δ::HIS3* [pRS315-pup1-T(-2)V]	this study
YWH231	*MAT***a** *his3-11,15 leu2-3,112 ura3 pre2Δ::HIS3* [pRS315-pre2-T1A]	this study
YWH232	*MAT***a** *his3-11,15 leu2-3,112 ura3 pre2Δ::HIS3* [pRS315-pre2-T1A-K81R]	this study
YEH73	*MAT***a** *his3-11,15 leu2-3,112 ura3 pre2Δ::HIS3* [pRS315-pre2-H(-2)L-T1A]	this study
YEH74	*MAT***a** *his3-11,15 leu2-3,112 ura3 pre2Δ::HIS3* [pRS315-pre2-H(-2)T-T1A]	this study
YEH75	*MAT***a** *his3-11,15 leu2-3,112 ura3 pre2Δ::HIS3* [pRS315-pre2-H(-2)A-T1A]	this study
YEH76	*MAT***a** *his3-11,15 leu2-3,112 ura3 pre2Δ::HIS3* [pRS315-pre2-H(-2)L-T1A-K81R]	this study
YEH77	*MAT***a** *his3-11,15 leu2-3,112 ura3 pre2Δ::HIS3* [pRS315-pre2-H(-2)T-T1A-K81R]	this study
YEH78	*MAT***a** *his3-11,15 leu2-3,112 ura3 pre2Δ::HIS3* [pRS315-pre2-H(-2)A-T1A-K81R]	this study

Strain	Genotype	Source
YWH2118	*MAT***a** *his3-11,15 leu2-3,112 ura3 pre2Δ::HIS3* [pRS315-UBI/mmPRE2+ppPRE2]	Jäger et al., 1999[7]
YWH2218	*MAT***a** *his3-11,15 leu2-3,112 ura3 pre2Δ::HIS3* [pRS315-UBI/mmpre2-D17N+ppPRE2]	this study
YWH2219	*MAT***a** *his3-11,15 leu2-3,112 ura3 pre2Δ::HIS3* [pRS315-UBI/mmpre2-D166N+ppPRE2]	this study
YWH2212	*MAT***a** *his3-11,15 leu2-3,112 ura3 pre2Δ::HIS3* [pRS315-UBI/mmpre2-T1A+ppPRE2] *nat1Δ::KanMX*	this study, *NAT1* gene deletion in strain YWH212 (Jäger et al., 1999)[7]
YWH203	*MAT***a** *his3-11,15 leu2-3,112 ura3 pre2Δ::HIS3* [pRS315-UBI/mmpre2-K33A+ppPRE2]	Jäger et al., 1999[7]

Note, that for simplicity the gene name *DOA3*, originally used in strains from the Hochstrasser lab, was replaced by *PRE2*. The numbering of amino acids deviates from that used for alleles described in Chen and Hochstrasser, 1996 and Heinemeyer et al., 1997, and refers to the processed β5 subunit (residues in the propeptide are preceded by a minus)[2].
mm = mature moiety, pp = propeptide

Supplementary Notes

Supplementary Note 1 Effects of mutations in β5 active site residues on yeast viability and proteasome function.

The T1A (formerly named T76A) mutation in the *PRE2/DOA3* gene encoding proteasome subunit β5 was in one case reported to be still compatible with yeast viability, although causing extremely slow growth (expression from YCplac22-*doa3-T1A* with 550 bp of promoter in the MHY501 strain background[4]); in another strain, however, it was found to be lethal (expression from pRS315-*pre2-T1A* with 370 bp of promoter in the WCG4 background[3]). To clarify this apparent difference, we re-sequenced both constructs and found an additional point mutation in the *doa3-T1A* allele that changed Lys81 in the mature β5 subunit to Arg81. After cloning this double mutant allele into pRS315 (with the same promoter length as in the pRS315 construct used in Heinemeyer et al., 1997[3]), plasmid shuffling resulted in viable, slow-growing cells in both strain backgrounds. We then tested the original pRS315-*pre2-T1A* construct again in both backgrounds, and we were able to isolate 5-FOA resistant clones in all cases, but colony formation on 5-FOA plates was retarded compared to the double mutant construct (4-5 versus 3 days). This might explain why viable strains with the T1A mutation in β5 were originally only found in context with the additional K81R mutation. After the selection step on 5-FOA the cells grow poorly with and without the K81R mutation indicating that the suppressing effect of this mutation is subtle and restricted to the outgrowth on 5-FOA medium. Our analysis clearly excludes any effects of the genetic background, the selection marker or the promoter length in the different plasmid constructs as explanations for the disparate results initially reported for the β5-T1A mutant.

In addition, the T1S (formerly named T76S) mutation in β5 had in one case been described to cause a severe growth defect and an almost complete failure in autocatalytic β5 precursor processing[4], whereas it caused a milder growth phenotype, slightly retarded maturation and no

reduction of β5 peptidase activity in the report of Heinemeyer et al., 1997[3]. Re-sequencing of the YCplac22-*doa3-T1S* allele reported in Ref. 1 suggested that this mutant construct was likely to have been mixed up with a plasmid carrying the *doa3-G(-1)A* (formerly named *doa3-G75A*) variant. The β5-T1S mutant does show a reduction in ChT-L peptidase activity and fails to grow at 37 °C in our current analysis (Fig. 4a, b and Supplementary Fig. 9a, c).

As with the T1A mutation, the K33A mutation (formerly named K108A) of subunit β5 initially was reported by our two groups to either prevent growth altogether[4] or still allow survival[3]. Our resequencing suggests that additional mutations in the allele that allowed survival are responsible for these differences: An L(-49)S change in the propeptide was found to be encoded in the *pre2-K33A* allele constructed by Heinemeyer et al., 1997[3], while the published *pre2-K33R* allele encodes an additional F(-45)S exchange in the β5 propeptide. Both double mutant alleles allowed survival in the MHY501 background as well, with cell proliferation being less retarded than for β5-T1A mutants. After removal of the propeptide mutations, β5-K33A or β5-K33R cells still yielded small viable colonies in both strain backgrounds after prolonged incubation on 5-FOA plates. After transfer from 5-FOA to YPD, the growth defects were more severe compared to clones harboring the additional propeptide mutations. The K33R mutation was slightly less growth inhibiting than the K33A mutation either with or without the additional propeptide mutations.

The D17N mutant of β5 (pp *cis*) has been created in both genetic backgrounds for the first time in the current work. It exhibited comparable or even more drastic growth defects than the T1A and K33A mutants and required several days of growth in large-scale cultures to obtain enough cells for proteasome purification, which, however, has so far been unsuccessful unless an additional propeptide mutation (L(-49)S) was introduced.

We are aware that for all viable but extremely impaired mutants with alterations of the catalytic β5 residues, various extragenic suppressor mutations are likely to have emerged when colonies were selected on 5-FOA plates. This leads to variations in the mutant defects,

but in all cases, there is a strong negative impact on growth, consistent with the paramount role of the β5 active site in the *in vivo* function of the proteasome.

Supplementary Note 2 The proteasomal substrate binding channel.

The proteasome bears the three proteolytically active subunits: β1, β2 and β5. Together with their adjacent subunits β2, β3 and β6, respectively, these entities form three non-primed substrate binding channels with distinct pockets (S1-S4), which accommodate peptide substrates for their subsequent endoproteolytic cleavage. The chemical nature of the amino acid at position 45 largely determines the character of the S1 site and hence substrate preferences of the three proteolytically active centers[2,8]. The hydrophobic Met45 in subunit β5 leads to chymotrypsin-like (ChT-L) activity, while Arg45 of subunit β1 promotes cleavage after acidic residues (caspase-like (C-L) activity) and Gly45 of subunit β2 enables rather broad substrate specificity with tendencies for basic residues (trypsin-like (T-L) activity).

Supplementary Methods

Yeast growth tests

Overnight cultures of WT (WCG4a and *pre2Δ::HIS3 [YCplac22-PRE2]*) and mutant yeast strains were grown in rich (YPD) medium at 30 °C. Their optical density was determined at 600 nm, and appropriate serial dilutions in YPD were prepared. Total cell numbers of 10,000, 1,000, 100 and 10 were spotted on YPD plates and grown for several days at 30 °C or 37 °C to evaluate proliferation rates by colony size as well as survival rates by colony numbers.

In situ test for chymotrypsin-like activity

Yeast strains, grown as patches on YPD plates, were replica plated onto sterile filter paper disks, which were then placed on fresh YPD plates and grown for an additional 2 days at

30 °C. Cells were lysed by immersing the filter paper in 10 ml of chloroform for 15 min and subsequently covering the filter with 10 ml of overlay solution (1 % (w/v) agar, 50 mM Tris-HCl pH 8.0, 300 µl of 10 mM Z-Gly-Gly-Leu-pNA). After incubation for 3 h at 30 °C, the following incubations were carried out: 10 ml of 0.1 % (w/v) sodium nitrite solution in 1 M HCl for 5 min; 10 ml of 0.5 % (w/v) ammonium sulfamate solution in 1 M HCl for 5 min; 10 ml of 0.05 % (w/v) *N*-(1-naphthyl)ethylenediamine solution in 47 % (v/v) ethanol for 10 - 30 min.

Substrate and inhibitor stock solutions

Peptide substrates (Bachem, Switzerland), bortezomib (Selleck Chemicals Ltd., USA), carfilzomib (Active Biochemicals Co. Ltd., China), MG132 (Selleck Chemicals Ltd., USA) were dissolved in DMSO and stored as stock solutions at - 20 °C.

Supplementary References

1. Huber, E.M., Heinemeyer, W. & Groll, M. Bortezomib-Resistant Mutant Proteasomes: Structural and Biochemical Evaluation with Carfilzomib and ONX 0914. *Structure* **23**, 407-17 (2015).
2. Groll, M. et al. Structure of 20S proteasome from yeast at 2.4 Å resolution. *Nature* **386**, 463-71 (1997).
3. Heinemeyer, W., Fischer, M., Krimmer, T., Stachon, U. & Wolf, D.H. The active sites of the eukaryotic 20 S proteasome and their involvement in subunit precursor processing. *J. Biol. Chem.* **272**, 25200-9 (1997).
4. Chen, P. & Hochstrasser, M. Autocatalytic subunit processing couples active site formation in the 20S proteasome to completion of assembly. *Cell* **86**, 961-972 (1996).
5. Li, X., Li, Y., Arendt, C.S. & Hochstrasser, M. Distinct Elements in the Proteasomal beta5 Subunit Propeptide Required for Autocatalytic Processing and Proteasome Assembly. *J. Biol. Chem.* (2015) jbc.M115.677047.
6. Heinemeyer, W., Gruhler, A., Mohrle, V., Mahe, Y. & Wolf, D.H. PRE2, highly homologous to the human major histocompatibility complex-linked RING10 gene, codes for a yeast proteasome subunit necessary for chymotryptic activity and degradation of ubiquitinated proteins. *J. Biol. Chem.* **268**, 5115-20 (1993).
7. Jäger, S., Groll, M., Huber, R., Wolf, D.H. & Heinemeyer, W. Proteasome beta-type subunits: unequal roles of propeptides in core particle maturation and a hierarchy of active site function. *J. Mol. Biol.* **291**, 997-1013 (1999).
8. Huber, E.M. et al. Immuno- and constitutive proteasome crystal structures reveal differences in substrate and inhibitor specificity. *Cell* **148**, 727–738 (2012).
